# miRNA analysis with *Prost!* reveals evolutionary conservation of organ-enriched expression and post-transcriptional modifications in three-spined stickleback and zebrafish

**DOI:** 10.1038/s41598-019-40361-8

**Published:** 2019-03-08

**Authors:** Thomas Desvignes, Peter Batzel, Jason Sydes, B. Frank Eames, John H. Postlethwait

**Affiliations:** 1 Institute of Neuroscience, University of Oregon, Eugene, OR 97403 USA; 2 Department of Anatomy, Physiology, and Pharmacology, University of Saskatchewan, Saskatoon, SK S7N 5E5 Canada

## Abstract

MicroRNAs (miRNAs) can have organ-specific expression and functions; they can originate from dedicated miRNA genes, from non-canonical miRNA genes, or from mirror-miRNA genes and can also experience post-transcriptional variation. It remains unclear, however, which mechanisms of miRNA production or modification are organ-specific and the extent of their evolutionary conservation. To address these issues, we developed the software *Prost!* (PRocessing Of Short Transcripts), which, among other features, helps quantify mature miRNAs, accounts for post-transcriptional processing, such as nucleotide editing, and identifies mirror-miRNAs. Here, we applied *Prost!* to annotate and analyze miRNAs in three-spined stickleback (*Gasterosteus aculeatus*), a model fish for evolutionary biology reported to have a miRNome larger than most teleost fish. Zebrafish (*Danio rerio*), a distantly related teleost with a well-known miRNome, served as comparator. Our results provided evidence for the existence of 286 miRNA genes and 382 unique mature miRNAs (excluding *mir430* gene duplicates and the vaultRNA-derived *mir733*), which doesn’t represent a miRNAome larger than other teleost miRNomes. In addition, small RNA sequencing data from brain, heart, testis, and ovary in both stickleback and zebrafish identified suites of mature miRNAs that display organ-specific enrichment, many of which are evolutionarily-conserved in the brain and heart in both species. These data also supported the hypothesis that evolutionarily-conserved, organ-specific mechanisms may regulate post-transcriptional variations in miRNA sequence. In both stickleback and zebrafish, miR2188-5p was edited frequently with similar nucleotide changes in the seed sequence with organ specific editing rates, highest in the brain. In summary, *Prost!* is a new tool to identify and understand small RNAs, to help clarify a species’ miRNA biology as shown here for an important model for the evolution of developmental mechanisms, and to provide insight into organ-enriched expression and the evolutionary conservation of miRNA post-transcriptional modifications.

## Introduction

microRNAs (miRNAs) are small non-coding RNA molecules about 20-22 nucleotides long that control gene expression post-transcriptionally by repressing translation or inducing the decay of targeted messenger RNA transcripts (mRNAs)^1–3^. miRNAs participate in virtually all biological processes, including the control of cell specification, cell differentiation, organ development, and organ physiology^4–6^ as well as pathologies in humans and other animals^7–10^. miRNA genes also appear to be evolutionarily-conserved in number, sequence, and syntenies across metazoans^3,11–15^, but the evolutionary conservation of miRNA organ expression patterns remain incompletely understood.

Canonically, miRNA genes are transcribed into a primary transcript (pri-miRNA) that folds into a hairpin, from which the enzyme Drosha cleaves off the free 5′ and 3′ ends, thereby producing the precursor miRNA (pre-miRNA). In the case of clustered miRNAs, the pri-miRNAs can be monocistronic or polycistronic and can fold into the same number of hairpins as the number of miRNAs in the cluster. The pre-miRNA, which assumes a stem-loop hairpin conformation, is then exported into the cytoplasm where a second enzyme, Dicer, trims off the loop and releases a miRNA duplex^16,17^. One strand of the miRNA duplex is usually degraded, while the other strand loads into the RNA-Induced Silencing Complex (RISC), the effector of the miRNA regulation system. Once incorporated into the RISC, the miRNA drives the association of the enzymatic complex to specific mRNA transcripts by base pairing of the miRNA seed (nucleotides 2–8 from the 5′ end) to the targeted transcript’s 3′ UTR. The association of the RISC to the messenger RNA will either induce the decay of the transcript or prevent its translation, depending on pairing strength. Other pathways and other gene types can also produce miRNAs (e.g. miRNAs from Drosha- or Dicer-independent pathways, miRNAs produced by both DNA strands at the same locus (mirror miRNAs), lncRNAs, and snoRNAs^18–21^) and the most common alternative miRNA biogenesis pathway is the processing of miRtrons, which are miRNA hairpins originating from spliced introns of protein-coding genes^22,23^.

Besides originating from a variety of biogenesis pathways and gene types, miRNA sequence variations can arise post-transcriptionally, resulting in variations in size and nucleotide sequence; these variants are called isomiRs^2,24,25^. The most frequent post-transcriptional modification involves variations in length at the 3′ end of miRNAs. Length modifications at the 5′ end of miRNAs occur less frequently than at the 3′ end, perhaps because they cause a shift in the seed, which can modify the identity of targeted transcripts and thus can drastically change the miRNA’s function^26,27^. miRNA sequence variation can also occur due to post-transcriptional editing, in which ADAR (adenosine deaminase, RNA-specific) enzymes post-transcriptionally modify a nucleotide, usually an adenosine (A), into another base, usually an inosine (I)^28–30^. These post-transcriptional modifications have now been shown to be physiologically relevant^30–34^, but whether post-transcriptional editing occurs in a directed and regulated, organ-specific manner is still currently unknown.

The diversity of miRNAs, their variations, and the rapid expansion of small RNA sequencing reveal the need for small RNA analysis tools that can encompass the full diversity of gene origins and variations in miRNA sequences. Several bioinformatic tools are currently available to study miRNAs using small RNA sequencing datasets^35–41^. To study miRNA expression, some tools compare sequenced reads to annotated RNA sequences without aligning directly to a genome^38,42^. Many tools start by filtering reads that can readily be annotated as miRNAs and then report their expression, sometimes using a genomic reference. Other tools make use of genomic alignments and specialize in the discovery of novel miRNAs^36,43^ or the study of isomiRs^35,44^. These tools often perform well for their respective functions, but in many cases, lack transparency in their filtering and annotating algorithms, have few user-defined parameter choices that might help tune a user’s specific application, and/or lack the ability to inspect the entire small RNA dataset and omit sequences not already annotated as a miRNA. With increasing amounts of data and sequence read diversity, a more global approach was required to address sequencing output by analyzing every single read – even if it is not yet annotated – as a type of coding or non-coding RNA. In addition, analysis tools should give attention to read alignments on a genomic reference to differentiate fragments potentially originating from one or multiple loci. While many tools are available to study small RNA sequencing datasets, current tools usually do not provide a comprehensive, genome-based analysis of small RNA datasets, thus limiting the study of the full complexity of an experiment by failing to report some of the post-transcriptional processes affecting the diversity of small RNAs.

To help study the complexity and diversity of miRNA sequences in small RNA-seq data, we generated a new software tool *Prost!* (PRocessing Of Small Transcripts) that facilitates the identification of miRNAs for annotation, quantifies annotated miRNAs, and details variations (isomiRs) observed in each sample. *Prost!* is open-source, publicly available software^45^. Earlier versions of *Prost!* have been used to annotate zebrafish and spotted gar miRNAs^46,47^, as well as to identify erythromiRs in white-blooded Antarctic icefish^48^.

To investigate the evolutionary conservation of miRNAs in teleost fish, we performed small RNA sequencing on four organs (brain, heart, testis, and ovary) in two distantly related teleost laboratory model fishes: the medical genetics model zebrafish *Danio rerio* and the evolutionary genetics model three-spined stickleback *Gasterosteus aculeatus*. While zebrafish miRNAs are well annotated^46,49,50^, stickleback miRNAs aren’t, and current predicted annotations provide miRNA gene number estimates ranging from several hundred to well over a thousand genes^51–54^, which is more than four times the number of miRNA genes in zebrafish. In addition, no study has so far investigated the potential conservation of miRNA expression patterns across teleost fish species, or studied post-transcriptional modifications in teleost mature miRNAs. Here, we addressed the following questions: (1) Is the stickleback miRNome significantly larger than that in other teleost species as reported? (2) Do zebrafish and stickleback share organ-enriched expression of specific miRNAs? And (3) Do animals regulate post-transcriptional modifications to display organ-specificity and are organ-specific modifications shared by zebrafish and stickleback?

## Materials and Methods

### Origin of sampled fish

Four zebrafish individuals (*Danio rerio*, AB strain, two males and two females) were obtained from the University of Oregon Aquatic Animal Core Facility and four three-spined stickleback (*Gasterosteus aculeatus*, two males and two females) of a fresh water laboratory strain derived from Boot Lake, Alaska were obtained from Mark Currey in the W. Cresko Laboratory (University of Oregon). To limit biases that might arise from differences in culture, physiological state, and sampling conditions, animals of each species were raised under their respective optimal conditions of temperature (20 °C and 28.5 °C for stickleback and zebrafish, respectively), photoperiod (12/12 h light/dark for both species), and densities (one fish per four liters and 10 fish per liter for stickleback and zebrafish^55^, respectively). In addition, all animals were sexually mature adults that had been reproductively active for several months. All animals were handled in accordance with good animal practice as approved by the University of Oregon Institutional Animal Care and Use Committee (Animal Welfare Assurance Number A‐3009‐01, IACUC protocol 12‐02RA).

### RNA extraction and small RNA library preparation

Immediately following euthanasia by overdose of MS-222, fin clips, brains, heart ventricles, and testes were sampled from two male zebrafish and two male stickleback, and fin clips and ovaries were sampled from two female zebrafish and two female stickleback. All organs were dissected by the same person and extractions were processed identically. DNA was extracted from fin clips. Proteinase K was used to break open the cells, cell debris was then precipitated by centrifugation (10 minutes, 4 °C, 12,000 g), and the DNA extract was washed once with isopropanol and centrifuged (10 minutes, 4 °C, 12,000 g), followed by two 75% ethanol washes and centrifugation steps (10 minutes, 4 °C, 12,000 g), before resuspension of DNA in nuclease-free water. Both small and large RNAs from each individual organ were extracted using Norgen Biotek microRNA purification kit (Norgen Biotek, Thorold, ON, Canada) according to the manufacturer’s instructions. Using the small RNA extract fractions, for each male of each species, we prepared three individual libraries (brain, heart ventricle, and testis), and for each female of each species we prepared a single library (ovary). In total, 16 small RNA libraries were then prepared and barcoded using the Bioo Scientific NEXTflex^TM^ small RNA sequencing v1 kit (Bioo Scientific, Austin, TX, USA) with 15 PCR cycles. Libraries were sequenced on the Illumina HiSeq 2500 platform at the University of Oregon Genomics and Cell Characterization Core Facility (GC3F). Raw single-end 50-nt long reads were deposited in the NCBI Short Read Archive under project accession numbers SRP157992 and SRP039502 for stickleback and zebrafish, respectively.

### *Prost!* workflow

Raw reads from all sixteen libraries were pre-processed identically. Reads that did not pass Illumina’s chastity filter were discarded. Adapter sequences were trimmed from raw reads using cutadapt^56^ with parameters:--overlap 10 -a TGGAATTCTCGGGTGCCAAGG --minimum-length 1. Reads were then quality filtered using fastq_quality_filter of the FASTX-Toolkit (http://hannonlab.cshl.edu/fastx_toolkit/commandline.html) (with parameters: −Q33 −q 30 −p 100). Remaining reads were converted from FASTQ format to FASTA format.

Filtered reads were processed using *Prost!*, which is available online at https://prost.readthedocs.io and https://github.com/uoregon-postlethwait/prost ^45^. Briefly, *Prost!* size-selects reads for lengths typical of miRNAs and tracks the number of reads matching any given sequence. For miRNAs, we configured *Prost!* to select for reads 17 to 25 nucleotides in length. *Prost!* then aligns the unique set of sequences to a reference genome using bbmapskimmer.sh of the BBMap suite (https://sourceforge.net/projects/bbmap/) (with parameters: mdtag = t scoretag = f inserttag = f stoptag = f maxindel = 0 slow = t outputunmapped = f idtag = f minid = 0.50 ssao = f strictmaxindel = t usemodulo = f cigar = t sssr = 0.25 trimreaddescriptions = t secondary = t ambiguous = all maxsites = 4000000 k = 7 usejni = f maxsites2 = 4000000 idfilter = 0.50). We configured *Prost!* to use the publicly available genome assemblies for three-spined stickleback (BROAD S1) and zebrafish (GRCz10)^54^ for the study of stickleback and zebrafish reads, respectively. *Prost!* then groups small RNA sequences that have overlapping genomic locations on each respective genome assembly. We configured *Prost!* to retain only sequences with a minimum of five identical reads for the initial annotation pass, and only sequences with a minimum of 30 reads for the differential expression analysis.

*Prost!* annotates reads grouped according to genomic location by aligning against the mature and hairpin sequences of known miRNAs using bbmap.sh of the BBMap suite, as well as by performing a reverse alignment of known mature sequences against the unique set of reads (with parameters: mdtag = t scoretag = f inserttag = f stoptag = f maxindel = 0 slow = t outputunmapped = f idtag = f minid = 0.50 ssao = f strictmaxindel = t usemodulo = f cigar = t sssr = 0.25 trimreaddescriptions = t secondary = t ambiguous = all maxsites = 4000000 k = 7 usejni = f maxsites2 = 4000000 idfilter = 0.50 nodisk). In the current study, we configured *Prost!* to use all mature and hairpin sequences for chordates in miRBase Release 21^49^, as well as the predicted stickleback miRNA annotations^51,53,54^, the extended zebrafish miRNA annotation^46^, and the spotted gar miRNA annotation^47^. All annotation datasets used in this study are available on the *Prost!* Github page (https://github.com/uoregon-postlethwait/prost). Gene nomenclature follows recent conventions^2^, including those for zebrafish^57^. For miRNA genes that didn’t display phylogenetic conservation and were only predicted by one study^51,53,54^ following criteria for confident annotation proposed by previous studies^2,49,50^, each miRNA was annotated if (1) it originated from a maximum of six loci on the genome (which is the maximum number of locations that members of the largest miRNA families yet known originate from, i.e. let7a-5p and miR9-5p); (2) both strands of the hairpin were present in the sequencing dataset; (3) it displayed consistent 5′-end processing; and (4) it had a minimal expression level of 5 RPM for at least one of the two strands. For each mature miRNA locus, the most expressed isomiR was retrieved and used as the reference in the annotation. The annotation strategy used is detailed in a specific file on the *Prost!* documentation page (https://prost.readthedocs.io). Supplementary Table 1 provides a description of the sequencing depth and annotation statistics for each library.

### Manual miRNA annotation

For miRNA genes known in several teleost species but absent from our sequencing data, we performed a manual search in the stickleback genome assembly BROAD S1. We first retrieved the precursor sequence deposited in miRBase Release 21^49^ for the gene in the most closely related teleost fish, and aligned the sequence to the stickleback genome assembly using BLASTN with sensitivity set for “short sequences” using Ensembl^54^. Candidate regions (E-val < 1), were declared to have conserved syntenies by comparing three genes upstream and three genes downstream of the genomic hit in the stickleback genome to the corresponding region in other species in which the miRNA was annotated. If Ensembl called one or more of the six flanking genes as orthologs, synteny was considered to be conserved, the gene was manually annotated in stickleback, and the precursor sequence was extracted from the stickleback genome assembly.

### Differential expression analyses

From *Prost!* output, we used the non-normalized read counts of annotated miRNA reads to perform differential expression analysis between organs by pair-wise comparisons using the DESeq2 package^58^. For isomiR reads predicted to be variants of two or more miRNAs with equal probability, we partitioned their read counts proportionally based on counts of unambiguously annotated miRNAs that might give rise to the isomiR. In addition, when expression of an annotated miRNA was not detected in an organ, a read count of one was used instead of zero to facilitate the calculation of an adjusted p-value for that miRNA. We selected the “local” type trend line fitting model (FitType) and, to avoid false positives, at the expense of potential false negatives, we used a stringent maximum adjusted p-value of 1% (Benjamini and Hochberg procedure to adjust for multiple testing) to consider miRNAs as differentially expressed between two organs. Each pairwise comparison was subsequently verified for appropriate p-value distributions and compatibility with the negative binomial probability model used by DESeq2 (Supplementary File 1 for stickleback and Supplementary File 1 for zebrafish). Heat maps were generated using the Broad Institute Morpheus webserver^55,59^ (https://software.broadinstitute.org/morpheus/) using log2-transformed normalized counts from annotated miRNAs that displayed a minimum normalized average expression of 5 Reads-per-Million (RPM) over the entire dataset. Hierarchical clustering on both rows and columns was performed using the “one minus Pearson’s correlation” model and the “average” linkage method.

### Organ-Enrichment Index

To evaluate organ-specific expression enrichment, we calculated for each miRNA an organ enrichment index (OEI), which is analogous to the tissue specificity index (TSI) ‘tau’ for mRNAs^60^ and has been previously used for miRNAs^61^. The OEI varies from 0 to 1, with OEI close to 0 corresponding to miRNAs expressed in many or most organs at similar levels (i.e. ‘housekeeping’ miRNAs), and OEI close to 1 corresponding to miRNAs expressed in a specific organ (i.e. organ-enriched miRNAs). The OEI for miRNA *j* is calculated as:

$$oe{i}_{j}=\frac{{\sum }_{i=1}^{N}\,(1-{x}_{j,i})}{N-1}$$

where *N* corresponds to the total number of organs studied and *x*_*i.j*_ is the expression of miRNA *j* in organ *i* normalized by the maximal expression among all organs.

### PCR analyses and target predictions

To confirm miRNA editing events, we designed PCR primers to amplify primary miRNAs (pri-miRNAs) both from genomic DNA and from large RNA extracts of each investigated individual. This process allows the verification of putatively edited bases, rules out single nucleotide polymorphisms (SNPs) with respect to the reference genome sequence, and tests whether the transcribed pri-miRNA contains the edited base. Supplementary Table 2 contains primer sequences. PCR reactions were performed as previously described^62^, and the product of each reaction was cleaned using Diffinity RapidTip (Diffinity Genomics, USA) and sequenced by Genewiz (South Plainfield, NJ, USA). The relative frequency of each base at various positions in each miRNAs was displayed using the WebLogo3 webserver^63^. Putative miRNA targets were predicted using miRAnda 3.3a^64,65^ with default parameters (i.e. -sc 140.0 -en 1.0) and the 3′ UTR longer than 24 nucleotides present in Ensembl release 79 genome assemblies (BROAD S1 for stickleback, Zv9 for zebrafish). Stickleback to zebrafish gene orthology was called by taking the ortholog with the lowest accession number as called by Ensembl Biomart.

## Results and Discussion

### *Prost!*, a tool for analyzing small RNA sequencing reads

**Figure 1:**
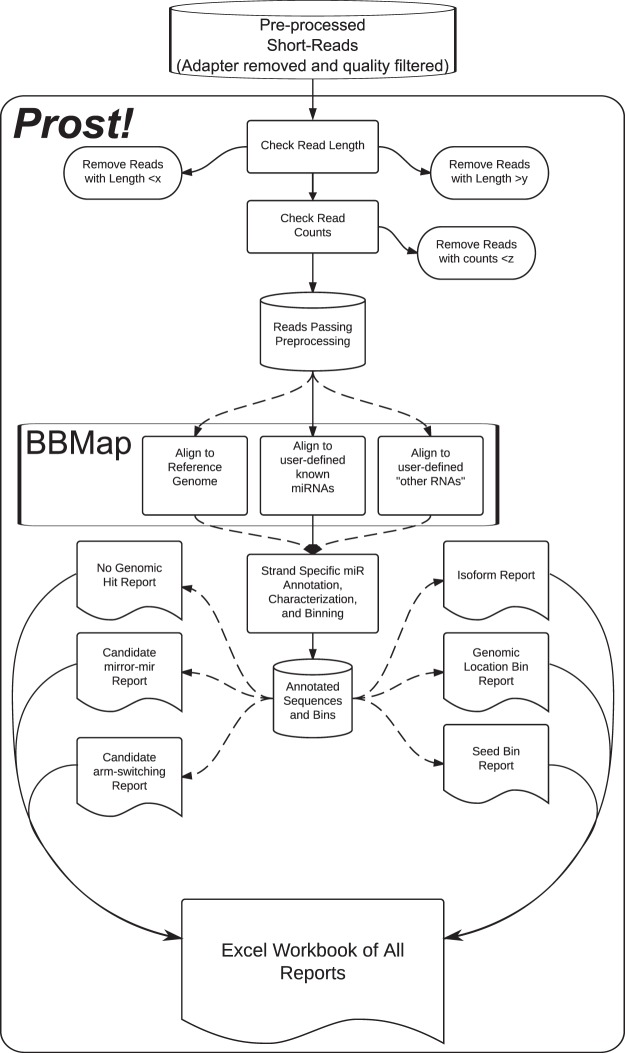
*Prost!* data processing flowchart. Flow chart displaying the input, pre-processing, categorization, alignment, and output report steps of *Prost!*

*Prost!* differs in three main ways from the majority of other tools developed to investigate small RNA-seq data. First, *Prost!* aligns reads to a user-defined genomic dataset (e.g. a genome assembly, Fig. 1). This initial alignment permits retention of all sequencing reads that match, perfectly or with a few errors, the “genomic dataset”, even if these matches are not yet known to be coding or non-coding RNA fragments. As such, *Prost!* enables the study of not only miRNAs, but also the identification of other small RNAs, such as piRNAs, t-RNA fragments, or the degradome of other RNA biotypes (e.g. snoRNAs, Y_RNA, vault-RNA). Second, *Prost!* groups reads based on their potential genomic origin(s), on their seed sequence, and ultimately on their annotation (Fig. 1). This step allows the regrouping of sequence variants that could originate from one locus or from multiple loci. Conversely, this step discriminates reads that could originate only from a limited number of paralogous loci, increasing the understanding of gene expression and locus-specific expression levels. Third, *Prost!* analyzes in depth the subset of reads that had been annotated based on the user-provided annotation dataset (e.g. miRNA or piRNA) and reports frequencies of individual sequence variations with respect to both the reference genome and the most expressed sequence that aligns perfectly to the genome from a genomic location group or annotation group (Fig. 1). This step ultimately provides a comprehensive report on potential post-transcriptional modifications for each group of sequences.

*Prost!* was written in Python and takes as input a list of sequencing sample files. *Prost!* can be configured with a simple and well annotated configuration file and optional command line flags, allowing the user to optimize *Prost!* for each specific dataset, experimental design, and experimental goal (e.g. annotation or quantification). The output can be retrieved either as an individual report per analysis step as tab-separated value files, or a single combined Excel file with each step provided as an individual tab that contains indexes, similar to primary-foreign keys of relational databases, facilitating the navigation from tab-to-tab to retrace and understand the entire analysis process (Fig. 1). Documentation on the *Prost!* Github page provides a complete description of the output file (https://prost.readthedocs.io). Supplementary Files 3 and 4 are *Prost!* output files used for differential expression analysis for stickleback and zebrafish, respectively.

### Stickleback miRNome

ZooMir^51^, Ensembl^54^, and Rastorguev *et al*.^53^ predicted that stickleback has 483, 504, and 595 miRNA genes respectively, and Chaturvedi *et al*.^52^ predicted 1486 mature miRNAs. Other well annotated teleost fish have substantially smaller miRNomes, consisting of about 250–350 genes^49,50,54^. This discrepancy raises the question of whether the stickleback miRNome is comparable to other well-annotated teleost genomes and contains approximately 250 to 300 miRNA genes, or whether the additional predicted stickleback miRNA genes are lineage-specific miRNAs and/or false predictions.

Using *Prost!* on our small RNA sequencing data of brain, heart, testis and ovary, we annotated 273 miRNA genes in stickleback with a total of 382 unique mature miRNAs (excluding the highly replicated *mir430* genes and the vaultRNA-derived *mir733*) (Table 1). Among these 273 miRNA genes, we were able to annotate both 5p and 3p strands for 221 genes (81%) and only one strand for 52 genes (19%) (Table 1). Among these 273 miRNA genes, three genes (*Rastorguev-366*, -458, and *-443*^53^, see Supplemental Files 5 and Table 3 for sequences) had reads in our sequencing data, but none of them displayed phylogenetic conservation. These three miRNAs are therefore likely to be stickleback-specific miRNAs. The manual annotation of known conserved teleost miRNAs^46,49,50^ that were not among the 273 stickleback miRNA genes annotated with *Prost!* identified 13 more miRNA genes (Table 1). For these 13 predicted miRNA genes, however, no mature miRNAs were present in our four-organ sequencing data, so we only annotated the putative pre-miRNAs.

**Table 1:** Stickleback miRNA annotation statistics.

Number of miRNA genes	286
Number of *mir430* genes (predicted by Ensembl)	139
Number of vaultRNA genes producing vault-derived miRNAs (i.e. *mir733* genes)	3
Total number of miRNA-producing genes	428
Number of mature miRNAs	500
Number of unique miRNAs	386
Number of miRNA genes with both 5p and 3p strands annotated	221 (77%)
Number of miRNA genes with only the 5p mature strand annotated	36 (13%)
Number of miRNA genes with only the 3p mature strand annotated	16 (6%)
Number of predicted miRNA genes with no mature strand annotated	13 (4%)

In partial summary, with *Prost!* and additional manual annotation, 286 miRNA genes were annotated in stickleback. This collection represents a miRNAome similar in size to other well-annotated teleost species that typically contain approximatively 250 to 300 miRNA genes (excluding the *mir430* genes). Supplementary Table 3 displays names, sequences, Ensembl Accession numbers if available, and positions on the stickleback ‘BROAD S1’ genome assembly for stickleback pre-miRNAs and mature miRNAs. Supplementary Files 5 and 6 provide FASTA format annotation for pre-miRNAs and unique mature miRNAs, respectively.

**Figure 2:**
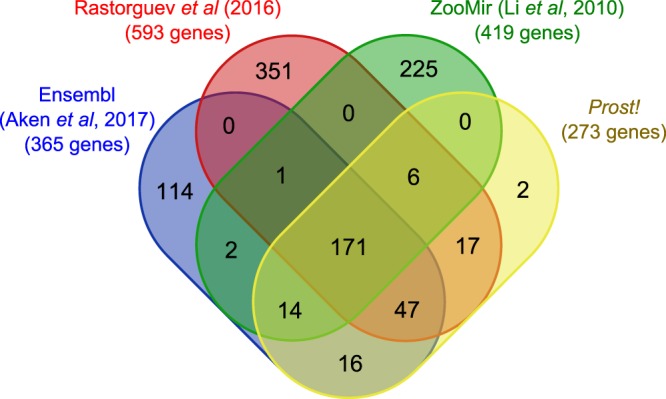
Overlap of several existing stickleback miRNA annotations. Genomic locations of stickleback pre-miRNAs were retrieved from other stickleback annotations^47,49,50^ and compared with each other and with those identified by *Prost!* A pre-miRNA from one annotation was considered the same as a pre-miRNA from another annotation if they shared at least a 25 nucleotide overlap.

By comparing all available annotations and by excluding *mir430* genes and the vaultRNA-derived *mir733* miRNAs that form unique large families of miRNA genes, we found that the stickleback annotation generated using *Prost!* contained many of the miRNAs found in other stickleback annotations^51,53,54^, lacked some other genes, and contained two additional genes not present in previous annotations. We did not include in our comparison the Chaturvedi *et al*.^52^ annotation because it was generated without strand-specificity. Our annotation included 194 of 419 (46%) miRNA genes in ZoomiR, 242 of 593 miRNA genes (41%) in Rastorguev *et al*., and 251 of 365 miRNA genes (69%) in Ensembl (Fig. 2), after excluding one, 64, and 139 *mir430* genes from Rastorguev *et al*., ZooMir, and Ensembl, respectively. The vaultRNA-derived *mir733* miRNAs were not present in any other annotations. Only three miRNAs were missing in our annotation that were present in at least two of the three other annotations, but all three of these (*mir204a*, *mir705*, and *mir1788*) were among the 13 known evolutionarily-conserved miRNAs that we annotated by orthology and for which no sequencing reads were present in our dataset. In addition, our annotation contained two genes that are absent from all other annotations. These two genes are *mir3120* (the mirror-miRNAs of *mir214*, see following section) and the ortholog of the zebrafish *mir723b* gene; both genes were previously annotated in zebrafish^46^. All other miRNAs missing from our annotation were predicted in only one of the other three annotations (Fig. 2) and our expression data couldn’t confirm their predictions according to confident annotation criteria defined previously^2,50^. *Prost!* thus provides conservative results in the annotation of miRNA sequencing data. This finding suggests that predicted miRNAs not found in our annotation either correspond to false predictions or are stickleback-specific genes that our sequencing data lacks because we explored just four tissues at one developmental stage. Sequencing of a larger diversity of tissues, developmental stages, and/or greater sequencing depth could, however, provide evidence of consistent biogenesis and expression of some of these miRNAs, therefore validating them as miRNA genes.

### Identification of mirror-miRNAs in teleosts

In addition to the annotation of mature miRNAs and miRNA genes, *Prost!* facilitates the identification of mirror-miRNA candidates by automatically filtering small RNA reads that originate from opposite DNA strands at the same location in the genome^21,46,66^. Teleost fish have mirror-miRNAs and some are conserved across vertebrate species, including the conserved mirror-miRNA pair *mir214*/*mir3120* in human and zebrafish, and at least two other teleost mirror-miRNA pairs (*mir7547*/*mir7553* and *mir7552a*/*mir7552aos*)^46^. In the list of candidate mirror-miRNAs generated by *Prost!*, we found the *mir214*/*mir3120* pair in both stickleback and zebrafish, but the two other known zebrafish mirror-miRNA pairs did not appear in our stickleback data. Although miR7552a-5p originating from the gene *mir7552a* was present in our stickleback sequencing data, sequencing reads from mirror *mir7552aos* were not; given the limited number of organs we studied, the mirror-miRNA pair *mir7552a*/*mir7552aos* might be expressed in other stickleback organs. The mirror-miRNA pair *mir7547*/*mir7553* was not only lacking from our sequencing data, but also was not found in the stickleback genome assembly by sequence homology or conserved synteny, providing no evidence for this miRNA pair in stickleback. Each of these three pairs of mirror-miRNAs, as well as potential new ones, might appear after sequencing a wider array of organs, more developmental stages, or with deeper sequencing. This analysis shows that *Prost!* readily confirmed the conservation of the mirror-miRNA pair *mir214*/*mir3120* in stickleback, demonstrating the genomic and transcriptomic conservation of these mirror-miRNAs among teleost fish.

### Organ-enriched miRNA expression

miRNAs are generally considered to be specialized in function and to display organ- and even cell type-specific enrichment^4,11,67^. Most of these data, however, are from mammals^61,68–70^, so the extent to which conservation of expression and function is similar among teleosts is unknown. We hypothesized that evolutionarily-conserved miRNAs should display organ-specific enrichment in stickleback and zebrafish.

**Figure 3:**
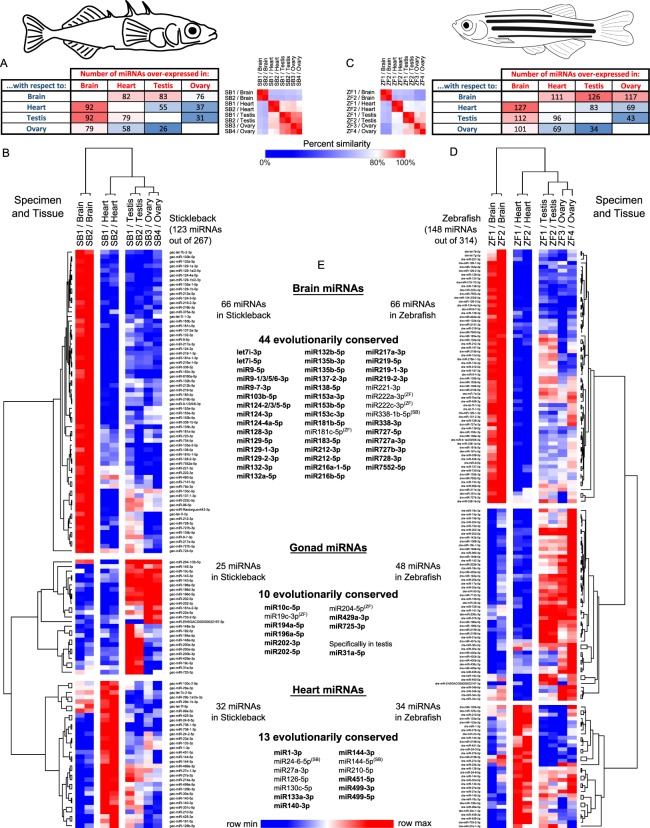
Differential expression and conservation of miRNAs in stickleback and zebrafish brain, heart, testis, and ovary. (**A**) Heat map showing the number of stickleback mature miRNAs over-expressed in each organ compared to each other organ along with a sample identity plot that compares the similarities for each of two samples of each organ to the other seven samples tested. (**B**) Heat map of the 123 stickleback mature miRNAs (in rows) that were consistently enriched in one organ (in columns) compared to the three other organs, or in gonads compared to brain and heart. (**C**) Heat map of the number of zebrafish mature miRNAs over-expressed in each organ compared to each other organ along with a sample identity plot. (**D**) Heat map of the 148 zebrafish mature miRNAs consistently enriched in one organ compared to the three other organs, or in gonads compared to brain and heart. For all heat maps, the deepest blue indicates the lowest level of expression in the row and the most intense red indicates the highest level of expression in the row. (**E**) Lists of organ-enriched miRNAs that are evolutionarily-conserved between stickleback and zebrafish. Bold lettering denotes that the miRNA has an OEI > 0.85 in both species. Superscripted SB (Stickleback) or ZF (Zebrafish) denotes that this specific miRNA has an OEI > 0.85 in the corresponding species but not in the other.

To investigate organ-specific enrichment of miRNA expression in stickleback, we studied the expression of 267 mature miRNAs (of the 321 that *Prost!* detected) that displayed an overall expression of at least 5 RPM across the entire dataset. Pairwise differential expression analysis using the DESeq2R package^58^ showed that (1) the brain displayed the greatest number of differentially expressed (DE) miRNAs among the four studied organs, and (2) the gonads (testis and ovary) displayed the fewest DE miRNAs and showed the largest intra-group variability (Fig. 3A).

In the six pairwise DE analyses of the four organs, 66 miRNAs were consistently over-expressed in the stickleback brain compared to each of the three other organs, compared to only 32, 10 and nine for heart, testis, and ovary, respectively (Fig. 3B, Supplementary Table 4). Because testis and ovary showed few organ-enriched DE miRNAs and share some common developmental processes in gametogenesis (e.g. meiosis and proliferation), we looked at miRNAs that were over-expressed in both testis and ovary compared to both heart and brain. Six additional miRNAs were similarly enriched in both testis and ovaries compared to the other organs, bringing the total number of miRNAs that are enriched in one or both gonads to 25 (Supplementary Table 4). Altogether, 123 miRNAs (i.e. 46% of the 267 minimally expressed miRNAs) displayed organ enrichment in either brain, heart, testis, ovary or in both gonads. This result validates the hypothesis that miRNAs in stickleback display organ-specific enrichment. More organ-enriched miRNAs would likely be identified after study of more organs.

**Figure 4:**
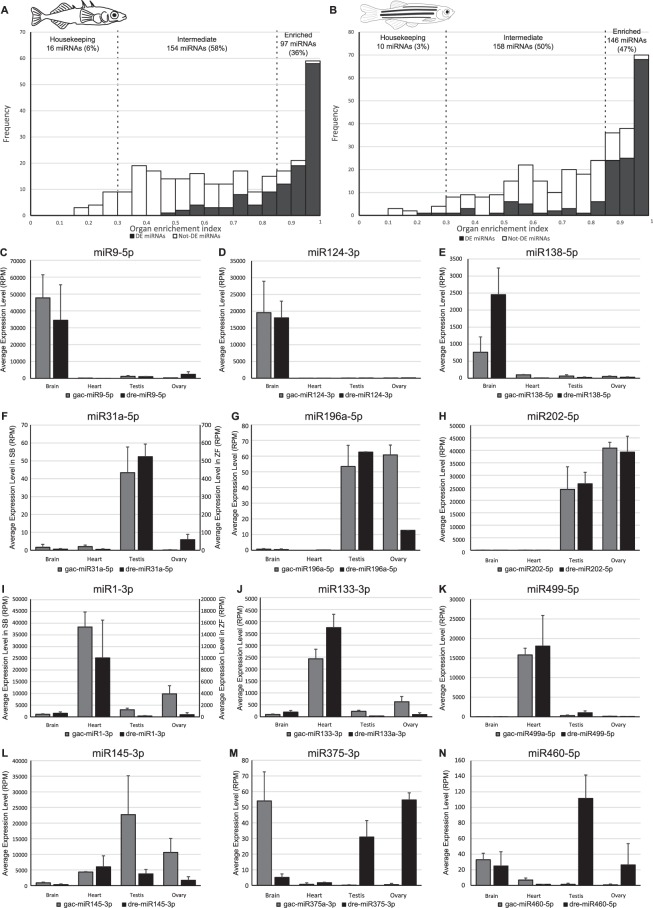
miRNA organ-enriched expression. (**A**,**B**) Frequency plot of OEI (organ enrichment index) values for all stickleback and zebrafish miRNAs expressed at more than 5 RPM across the entire dataset. Grey bars represent miRNAs that were also enriched in brain, heart, testis, ovary, or in both gonads, and white bars represent miRNAs that were not found to be enriched in a specific organ. (**C**–**N**) Average organ expression of evolutionarily-conserved, organ-enriched miRNAs that have an OEI > 0.85. Expression levels are given in RPM (Reads per Million) with associated standard deviations for the four organs studied in both stickleback (grey bars) and zebrafish (black bars).

To confirm this differential expression result, we analyzed the organ enrichment of each minimally expressed miRNA using the organ enrichment index (OEI), combining the testis and ovary data into a common ‘gonad’ organ type. Among the studied miRNAs, most (154/267 = 58%) displayed intermediate enrichment – they were predominantly expressed in one or more organs but were expressed significantly in at least one other organ (Fig. 4A). A total of 97 miRNAs (36%) showed an OEI > 0.85, which is considered a threshold for organ-enrichment^60,61^, and only 16 miRNAs (6%) showed ubiquitous, statistically similar expression levels among the organs studied with an OEI ≤ 0.3 (Fig. 4A). In addition, DE miRNAs tended to have the highest OEI scores (Figs 3B and 4A). Among the DE miRNAs that also had an OEI > 0.85, some displayed clear enrichment in brain (e.g. miR9-5p, miR124-3p, and miR138-5p, Fig. 4C–E), in testis (i.e. miR31a-3p, Fig. 4F), in both gonads (e.g. miR196a-5p and miR202-5p, Fig. 4G,H), or in heart (e.g. miR1-3p, miR133-3p, miR499-5p, Fig. 4I–K). Because we studied only four organs (and combined ovary and testis in the OEI analysis), some miRNAs that we categorized as not-organ-enriched might be enriched in organs that we didn’t study. For example, miR122-5p, which is known to be mostly expressed in liver in vertebrates^50,61^, showed low, non-specific expression in all four organs we investigated with an average of 18 RPM and an OEI score of 0.53.

To identify organ-specific enrichment of miRNAs in zebrafish, we studied the expression of 314 mature miRNAs (of the 402 that *Prost!* detected) that displayed an average expression of at least 5 RPM across all eight zebrafish samples. Similar to stickleback, the brain had the most differently expressed miRNAs, while ovary and testis had the least and showed the largest intra-group variability (Fig. 3C).

In all zebrafish pairwise comparisons, 66 miRNAs were consistently enriched in brain, 34 in heart, nine in testis, 21 in ovary, and an additional 18 miRNAs were equally enriched in both gonads (Fig. 3D, Supplementary Table 5). Altogether, 148 miRNAs (47% of the 314 minimally expressed zebrafish miRNAs) displayed organ-enrichment in brain, heart, testis, ovary, or in both gonads in zebrafish. Similar to the stickleback OEI analysis, of the 314 zebrafish miRNAs studied, most miRNAs (158/314 = 51%) displayed intermediate enrichment, 10 miRNAs (3%) showed overall ubiquitous expression levels among the studied organs, and 146 miRNAs (46%) showed organ-enrichment (OEI > 0.85) (Fig. 4A). Also similar to stickleback, we observed that miRNAs identified as organ-enriched by differential expression analyses are among the miRNAs that have the highest OEI (Figs 3D and 4B).

Taken together, these results demonstrated that a large proportion of miRNAs displayed enrichment in a single organ in both stickleback and zebrafish (46% and 47%, respectively) and organ-enrichment scores above 0.85 (36 and 46% in stickleback and zebrafish, respectively).

### Organ-enriched miRNAs are conserved between stickleback and zebrafish

The hypothesis that miRNA functions are conserved predicts that at least some of the organ-enriched miRNAs in stickleback would also be enriched in the same organ in zebrafish. To test this prediction, we compared the list of organ-enriched miRNAs in stickleback and zebrafish and found that 44 miRNAs were brain-enriched in both species (Fig. 3B,D,E), with many of them already known to be brain-associated miRNAs in several fish species^71–73^; for example miR9-5p, miR124-3p, and miR138-5p (Fig. 4C–E), which are also highly expressed in brain and nervous organs in mammals^50,61,74–78^. These observations suggest a strong evolutionary conservation of function for brain-related miRNAs among vertebrates. The heart also displayed a substantial number of evolutionarily-conserved, organ-enriched miRNAs (13 miRNAs) (Fig. 3B,D,E). Heart-enriched miRNAs included mature products of the well-described vertebrate cardiac myomiR genes *mir1*, *mir133*, and *mir499*^61,72,73,79–82^ and erythromiRs *mir144* and *mir451*^48^. The former group participates in muscle formation and function, and the latter may reflect the presence of red blood cells in the heart ventricle at the time of RNA extraction.

Surprisingly, gonad-enriched miRNAs appeared to be less conserved. Only one miRNA, miR31a-5p, was found to be testis-enriched in both stickleback and zebrafish (Figs 3B,D,E and 4F), while no miRNAs were ovary-enriched in both species (Fig. 3B,D,E). In chicken, *Mir31* has been hypothesized to be involved in gonadal sex differentiation because it is expressed significantly higher in testes compared to ovaries at early sexual differentiation stages^83^. In human, *MIR31* was down-regulated in the testis of an infertile adult human patient^84^. In fish, *mir31* has not yet been associated with either gonad differentiation or testicular function, but our data are consistent with a role for *mir31* that is conserved in testicular function among various vertebrate lineages. In addition, nine miRNAs were enriched in one or both gonads in both species (Fig. 3B,D,E), potentially reflecting a shared role in reproduction in one or both sexes in both species. Interestingly, among the gonad-associated miRNAs in stickleback and zebrafish, most displayed species-specific organ enrichment. For example, miR429a-3p was enriched in testis in stickleback but enriched in ovary in zebrafish; miR10c-5p was enriched in ovary in stickleback but enriched in testis in zebrafish; miR204-5p was enriched in ovary in zebrafish but enriched in both gonads in stickleback; miR196a-5p was enriched in testis in zebrafish but in both gonads in stickleback (Fig. 4G); and miR19c-3p, miR194a-5p, and miR725-3p were enriched in testis in stickleback but enriched in both gonads in zebrafish. In the case of the well-known gonad-enriched miR202-5p^85–87^, the expression level in the stickleback ovary was significantly higher than in testis; although the trend was the same in zebrafish, the difference was not statistically significant (Fig. 4H). The significance of these sex-specific differences is as yet unknown.

The relatively weak evolutionary conservation of sex-specific gonad enrichment in teleost fish is surprising and suggests reduced selective pressure on their function compared to other organ-enriched miRNAs, and/or that differences in the genetic control of reproduction exist between zebrafish and stickleback. Not enough information is currently available to distinguish between these two non-exclusive hypotheses. The large range of variations in sex-determination mechanisms, reproductive systems, reproductive state, and frequency of reproduction in teleost fish^88,89^, however, might help explain the weak conservation of stickleback and zebrafish miRNA expression in gonads. Indeed, the miRNA regulation system might be evolving with each species’ reproductive biology and its associated genetic regulation. Some ancestral functions of a miRNA could be conserved in one species, could have evolved novel targets and regulatory pathways in another, or may simply be lost in a lineage-specific fashion. For example, a gonad-enriched ancestral miRNA might have specialized in testis in one lineage, while remaining gonad-enriched or becoming ovary-enriched in another lineage, as could have happened with miR10c-5p or miR429a-5p in our data.

Only three other miRNAs displayed organ-enriched expression in different organs in zebrafish and stickleback: miR145-3p, miR375-3p, and miR460-5p were enriched in one organ in one species but in a different organ in the other species. miR145-3p was moderately enriched in heart in zebrafish, but was largely enriched in testes and moderately enriched in ovary in stickleback, while displaying similar levels of heart expression in both species. In zebrafish, miR375-3p and miR460-5p were strongly enriched in testis and in “gonads” (testis and ovary) but both were strongly enriched in brain in stickleback (Fig. 4M,N). The study of more teleost species, and in particular, the inclusion of an outgroup to represent the common ancestor, such as spotted gar, is necessary to test hypotheses regarding the loss-of-function, sub-functionalization, and neo-functionalization of miRNAs in teleost fishes.

### Evolutionarily-conserved, brain-enriched post-transcriptional miRNA seed editing

Post-transcriptional modification of miRNAs is frequent and generally originates from variation in biogenesis or enzyme-catalyzed nucleotide modification, generating groups of related sequences called isomiRs^2,24,25^. To study post-transcriptional modifications in teleost fish and to ask whether modifications are evolutionarily-conserved, we developed a feature in *Prost*! to calculate and color-code (in the Excel output) each type of post-transcriptional modification at individual genomic loci.

**Figure 5:**
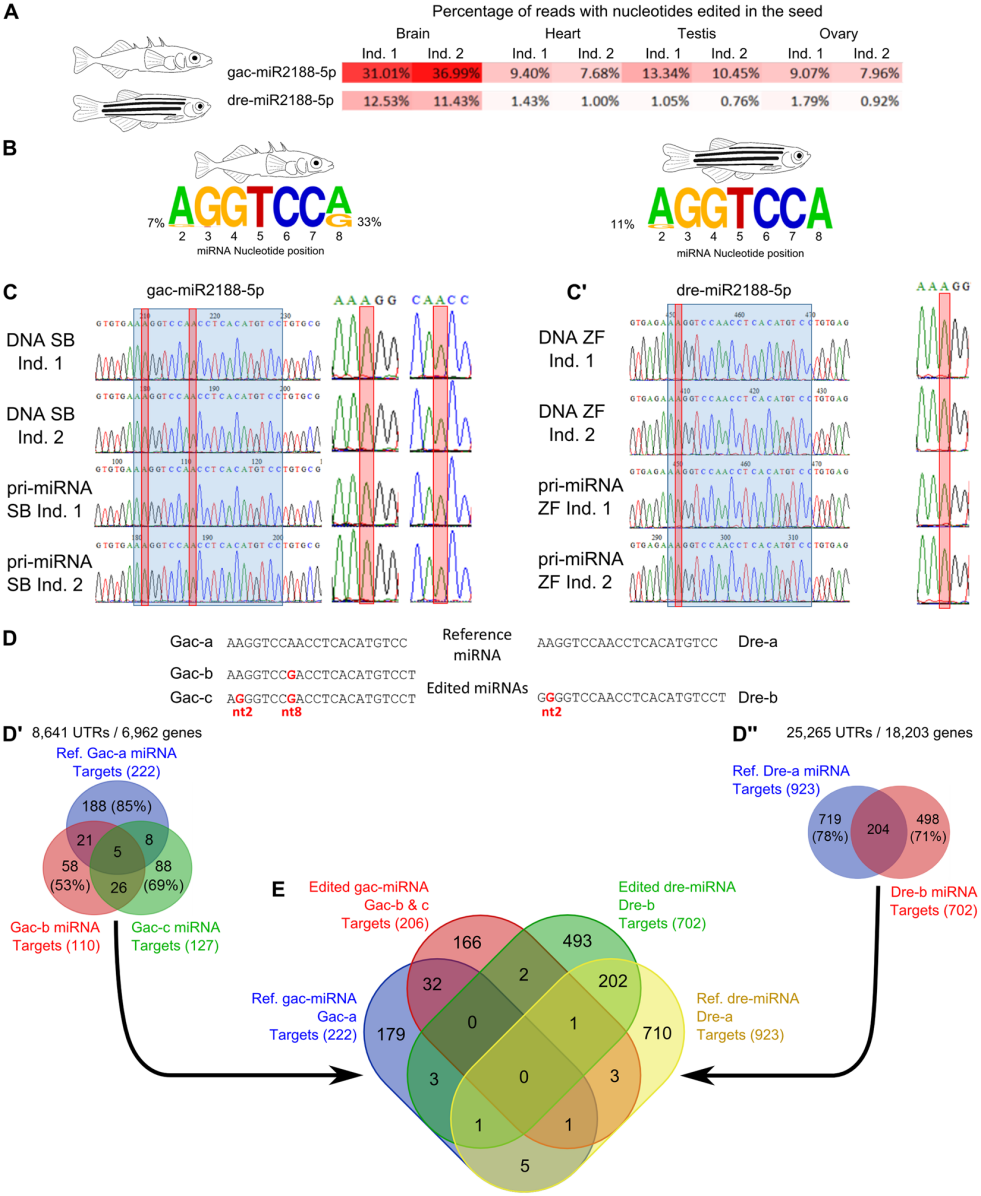
Evolutionarily-conserved brain-enriched seed-editing of miR2188-5p. (**A**) miR2188-5p seed editing frequency is higher in brain compared to other organs in both stickleback and zebrafish. (**B**) Frequency of seed variants generated using WebLogo3 webserver. (**C**) Sanger sequencing of genomic DNA and pri-miRNAs of both stickleback specimens. (C’) Sanger sequencing of genomic DNA and pri-miRNAs of both zebrafish specimens. The blue box highlights regions corresponding to mature miRNAs and red boxes highlight editing sites. (**D**) Mature miR2188-5p sequences used for target prediction in both stickleback and zebrafish. (D’) Overlap of the predicted target mRNA sets for each of the three mature miR2188-5p isomiRs in stickleback. (D”) Overlap of the predicted target mRNA sets for each of the two mature miR2188-5p isomiRs in zebrafish. (**E**) Overlap of predicted target mRNA sets for either genetically encoded or edited mature miR2188-5p sequences in both stickleback and zebrafish.

An analysis of overall read diversity and isomiR composition revealed that, in both stickleback and zebrafish, most isomiRs were 21 to 23 nucleotides long (Supplemental File 7A), and that the 10 most expressed miRNAs, including their respective isomiRs, accounted for more than 75% of total reads with most of these miRNAs common to both species (Supplemental File 7B). Furthermore, analysis of post-transcriptional modification types revealed that, in both species, approximately 35% of reads displayed 3′-end length variations (templated or non-templated), while only about 3.5% of reads displayed 5′-end length variations (Supplemental File 7C). This result is consistent with the fact that 5′-end length variations shift the seed sequence, which is likely to drastically alter the function of a miRNA by modifying the pool of its targeted mRNAs; in contrast, 3′-end length variations are less likely to strongly impact target recognition^2,26^. In addition, we observed that miRNA editing is rare in both species with, on average, 0.91% and 4.40% of reads displaying edition in the seven-nucleotide seed in stickleback and zebrafish respectively, and about 1.28% and 0.94% of reads displaying editing in the 15 nt or so outside of the seed in stickleback and zebrafish, respectively (Supplemental File 7C). Further automated analysis of *Prost!* seed-edition calculations revealed that in stickleback, miR2188-5p was prone to seed editing, especially in the brain (34%) compared to other organs (9.6%) (Fig. 5A). In zebrafish, similar analyses of post-transcriptional modifications revealed that the same miRNA, miR2188-5p, also displayed a higher rate of seed editing in the brain (12%), compared to other organs (1.0%; Fig. 5A). *Prost!* output also revealed that the stickleback miR2188-5p isomiR pool was composed of sequences displaying three different seeds: the genome-encoded seed (Gac-a in Fig. 5D; 67% of the sequences), a seed with an adenosine-to-guanosine (A-to-G) substitution at position 8 of the miRNA (Gac-b; 26% of the sequences), and a seed with two A-to-G substitutions, one at nucleotide 2 and one at nucleotide 8 (Gac-c; 7% of the sequences) (Fig. 5B). Examination of seed variations in zebrafish identified two different seeds: the genome-encoded seed (Dre-a in Fig. 5D; 89% of the sequences) and a seed with an A-to-G substitution at the second nucleotide of the mature miRNA (Dre-b; 11% of the sequences) (Fig. 5B). Because inosine bases are replaced by guanosine bases during the cDNA synthesis step of the preparation protocols for sequencing libraries, sequencers report a guanosine where an inosine could have originally been present in the RNA molecule. Therefore, nucleotides sequenced as guanosine in place of a genome-encoded adenosine in our sequencing data may have been due to be post-transcriptionally ADAR-edited inosine nucleotides.

To verify that the A-to-G substitutions we observed are due to post-transcriptional modification instead of potential miRNA allelic variations, we sequenced the *mir2188* gene and *pri-miR2188* transcript from each stickleback and zebrafish individual used for small RNA sequencing. In both species, the genomes of both individuals contained the same nucleotides at seed editing sites as found in the reference genome (Fig. 5C,C’). In addition, we found that the *pri-miR2188* was free of nucleotide substitutions at the second and/or eighth nucleotide of the mature miRNA in all individuals (Fig. 5C,C’). The genome sequencing results show that sequence variants in the miR2188-5p seed were not genetically encoded and the *pri-miRNA* sequencing result suggests that they occurred post-transcriptionally after the processing of the hairpin. We conclude that post-transcriptional seed editing of miR2188-5p is organ-enriched and evolutionarily-conserved. This finding represents, to our knowledge, the first example of teleost organ-enriched evolutionarily-conserved seed editing.

miRNA seed editing, by changing the seed sequence, can alter the set of targeted transcripts and therefore modify a miRNA’s function^30–34^. To evaluate the potential biological effects of miR2188-5p seed editing, we used miRAnda^64,65^ and 3′ UTR sequences of mRNAs to predict mRNA targets of both the reference miRNA and the edited miRNAs (Fig. 5D, Supplemental Table 6). For stickleback, the three different miR2188-5p isomiRs had few overlapping predicted target genes; in most cases, putative targets were unique to each isomiR (Fig. 5D’). For zebrafish, the targets predicted for each isomiR were also largely non-overlapping, with less than 15% of predicted targets in common for both isomiRs (Fig. 5D”).

To see if seed editing is likely to affect genes conserved between zebrafish and stickleback, we analyzed overlaps among putative mRNA target sets for miR2188-5p isomiRs in both species. Two, not mutually exclusive, hypotheses could explain the function of miRNA editing. Under one hypothesis, editing offers a new set of targets, and under the other hypothesis, miRNA editing removes targets from the list hit by the reference sequence. Among genes targeted by either the non-edited and/or the edited miRNAs in each species (394 and 1421 genes in stickleback and zebrafish, respectively), 309 genes displayed orthology relationship between stickleback and zebrafish based on Ensembl Biomart. Under the first hypothesis (gain of targets), results identified only two of 309 orthologs that were not predicted targets of the genomically encoded isomiR but were predicted targets of the seed-edited isomiRs in both stickleback and zebrafish. One of these two genes was *cntnap3* (*contactin associated protein like 3*, ENSDARG00000067824), a cell adhesion molecule of unknown function in teleost fish. In human and mouse, however, *CNTNAP3* is expressed in brain and spinal cord^90,91^, and its dysregulation in developing mice impairs motor learning and social behavior^92,93^. In addition, high CNTNAP3 levels have been associated with schizophrenia in humans^94^. The second conserved target of the seed-edited miR2188-5p is *pdha1a* (*pyruvate dehydrogenase alpha 1*, ENSDARG00000012387), which is strongly expressed in the brain of developing zebrafish embryos^95^. *PDHA1* mutations in human cause acid lactic buildup, resulting in impaired psychomotor development and chronic neurologic dysfunction with structural abnormalities in the central nervous system (OMIM 300502, ORPHA:79243).

Under the second hypothesis for the function of seed editing (removal of targets), only five genes were predicted to be targets of the genomically encoded isomiR but were not predicted targets of the seed-edited isomiRs in both stickleback and zebrafish. Among these five conserved targets of the genomically encoded, but not the edited miR2188-5p, four genes (*cyth1b*, *dcn*, *dcun1d1*, and *polr3e*) don’t seem to be involved in neuron or brain function in vertebrates. The fifth gene, *snap29* (synaptosome associated protein 29, ENSDARG00000038518), a soluble N-ethylmaleimide-sensitive factor-attachment protein receptor (SNARE), has also not yet been shown to be associated with brain phenotypes in zebrafish^57^. *SNAP29* mutations in human, however, cause a unique constellation of clinical manifestations including microcephaly, severe neurologic impairment, psychomotor retardation, referred as CEDNIK (cerebral dysgenesis, neuropathy, ichthyosis, and keratoderma) syndrome (OMIM 604202, ORPHA:66631).

The predicted conservation of potential targets urges functional analyses to study the effect of native or seed-edited miR2188-5p on brain transcript translation and its subsequent phenotypic consequences. To our knowledge, however, no prior publications have identified miR2188-5p as an edited miRNA nor suggested a role for it in brain function, arguing for more research. The study of additional species is needed to understand the phylogenetic conservation of the brain-enriched seed-editing of miR2188-5p and functional analyses are required to decipher a potential role in vertebrate brain development and physiology.

## Conclusions

Results reported here show that the novel software *Prost!* permitted the annotation of 273 miRNA genes in three-spined stickleback and that subsequent manual annotation annotated 13 additional genes. The stickleback miRNome, with a set of 286 miRNA genes, is thus comparable to the miRNome of other teleost species rather than being greatly enlarged as suggested by previous analyses^51–54^. *Prost!* analysis of small RNA sequencing libraries from more tissues or stages, however, is likely to permit the annotation of additional miRNA genes. In addition, as predicted, the differential expression analysis of miRNAs in four organs revealed significant organ-enrichment in either brain, heart, testis, ovary, or both gonads for about 46% and 47%of minimally expressed miRNAs in adult stickleback and zebrafish. Furthermore, supporting the hypothesis that organ-enriched miRNAs are evolutionarily-conserved, enriched expression of specific miRNAs was found in both brain and heart of both stickleback and zebrafish. In ovary and testis, however, fewer expressed miRNAs were conserved between these two teleosts, although several miRNAs that were enriched in both gonads of one species tended to be enriched in at least one of the two gonad types in the other species. Finally, we demonstrated the conservation of organ-specific miR2188-5p seed editing in the brains of both zebrafish and stickleback, suggesting potential conservation of this organ-specific, post-transcriptional seed editing process among teleosts.

## Supplementary information

Supplementary Datasets Description

Supplementary File 3

Supplementary File 4

Supplementary Table 3

Supplementary Table 4

Supplementary Table 5

Supplementary Table 6

## Data Availability

All data generated or analyzed during this study are included in this published article (and its Supplementary Information files) and raw Illumina sequencing reads were deposited in the NCBI Short Read Archive under project accession numbers SRP157992 and SRP039502 for stickleback and zebrafish, respectively.
