# Supplementary material for: miRNA analysis with Prost! reveals evolutionary conservation of organ-enriched expression and post-transcriptional modifications in three-spined stickleback and zebrafish
Source: Sci Rep. 2019 Mar 8;9:3913. doi: 10.1038/s41598-019-40361-8 (PMC6408482; doi:10.1038/s41598-019-40361-8)
Supplement: Supplementary file 1 — Supplementary Datasets Description [file 41598_2019_40361_MOESM1_ESM.docx]

# miRNA analysis with *Prost!* reveals evolutionary conservation of organ-enriched expression and post-transcriptional modifications in three-spined stickleback and zebrafish

Thomas Desvignes^1*#^, Peter Batzel^1*^, Jason Sydes^1*^, B. Frank Eames^2^, John Postlethwait^1^

^1^  Institute of Neuroscience, University of Oregon, Eugene OR 97403, USA

^2^ Department of Anatomy, Physiology, and Pharmacology, University of Saskatchewan, Saskatoon, SK S7N 5E5 Canada

* These authors contributed equally

# Correspondence to desvignes@uoneuro.uoregon.edu

# Supplementary Datasets

Supplementary File 1: Pairwise differential expression graphs in stickleback organs. *(Page 3)*

Supplementary File 2: Pairwise differential expression graphs in zebrafish organs. *(Page 4)*

Supplementary File 3: *Prost!* output file used for differential expression analysis for stickleback. *(Separate excel file)*

Supplementary File 4: *Prost!* output file used for differential expression analysis for zebrafish. *(Separate excel file)*

Supplementary File 5: Stickleback primary miRNA annotation. *(Page 5)*

Supplementary File 6: Stickleback mature miRNA annotation. *(Page 34)*

Supplementary File 7: miRNA isomiR diversity in stickleback and zebrafish. *(Page 53)*

Supplementary Table 1: small RNA library sequencing statistics. *(Page 53)*

Supplementary Table 2: PCR primers used for the study of miR2188-5p. *(Page 54)*

Supplementary Table 3: Complete stickleback miRNA annotation. *(Separate excel file)*

Supplementary Table 4: Organ-specific lists of differentially expressed miRNAs in stickleback. *(Separate excel file)*

Supplementary Table 5: Organ-specific lists of differentially expressed miRNAs in zebrafish. *(Separate excel file)*

Supplementary Table 6: Lists of predicted stickleback and zebrafish targets for the genomically encoded and edited isomiRs of miR2188-5p. *(Separate excel file)*

Supplementary File 1: Pairwise differential expression graphs in stickleback organs.


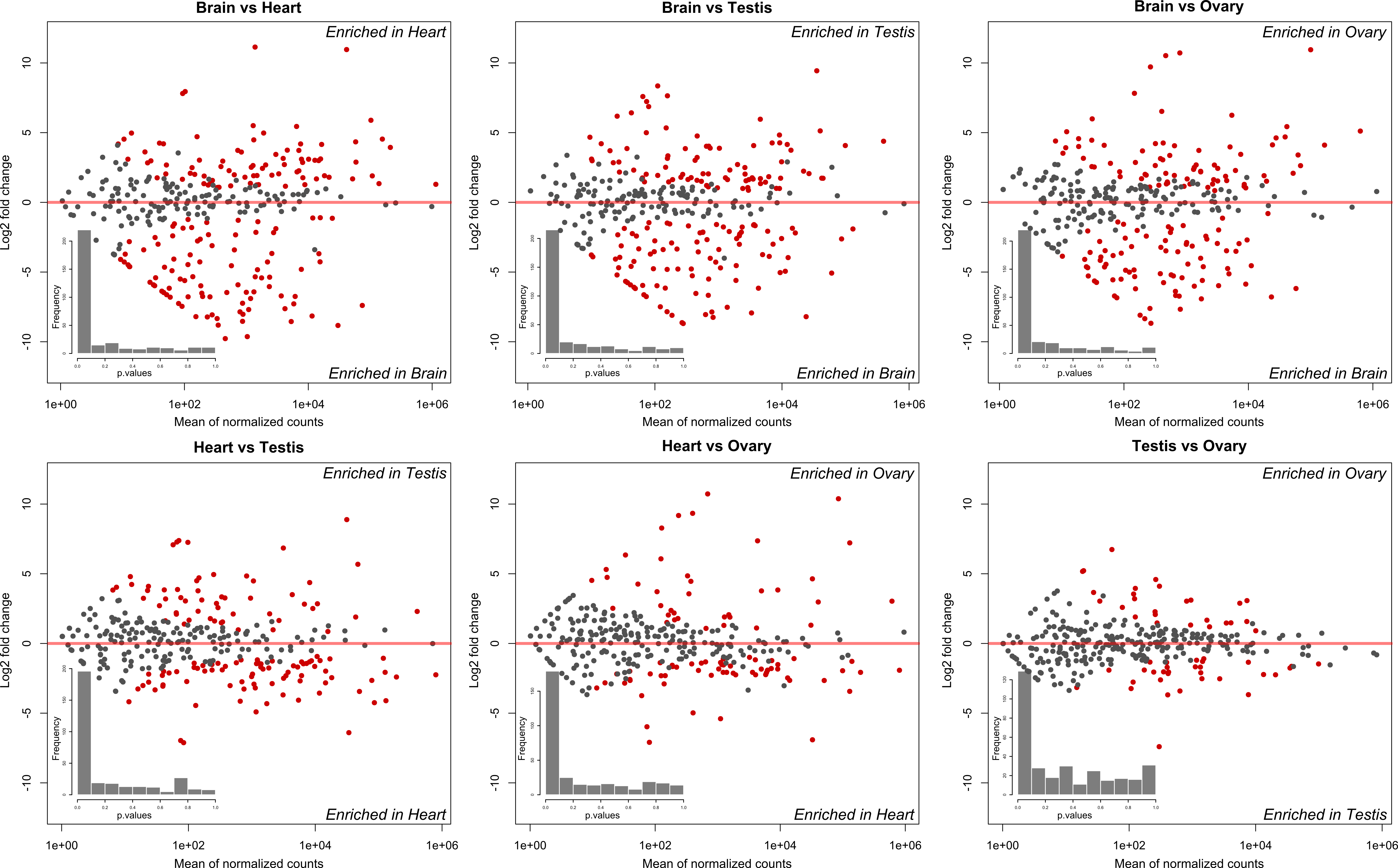


Supplementary File 2: Pairwise differential expression graphs in zebrafish organs.


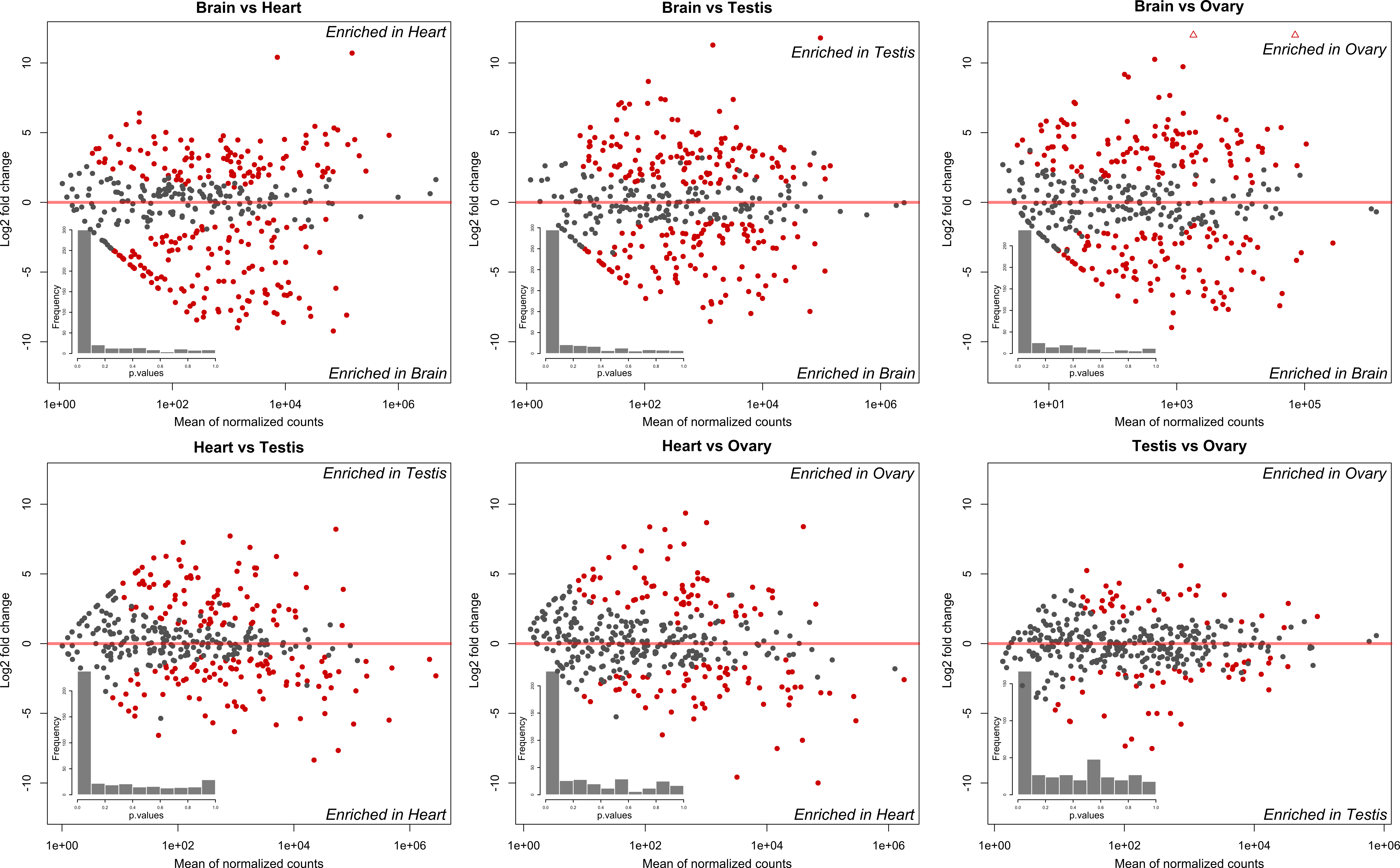


Supplementary File 5: Stickleback primary miRNA annotation.

>gac-let-7a-1

TGCACTGTGGGATGAGGTAGTAGGTTGTATAGTTTTACGGTCATACCCGCACTGGGAGATAACTATTAAACCTACTGTCTTTCCCAAAG

>gac-let-7a-3

ACGGCCCTTTGGGGTGAGGTAGTAGGTTGTATAGTTTTAGGGTCATTCCCAAGCTGTCAGATGACTATACAACTTACTGTCTTTCCTGAAGCGGCAGTAATGGC

>gac-let-7a-4

CAGGTTGAGGTAGTAGGTTGTATAGTTCAGAGTGACGCCACAGGAGATGACTGTACAGCCTCCTAGCTTTCCCT

>gac-let-7a-5

TTTGGGCTCTGTGAGGGTGAGGTAGTAGGTTGTATAGTTTGGTGGGTGGGATTGCACCCTGCTCCGGTGATAACTATACAGTCTATTGCCTTCCC

>gac-let-7a-6

CGAGGTGAGGTAGTAGGTTGTATAGTTTGTGGGATTGAGTAAATCCTACTCAGGTGATAACTACACAGCCTATTACCTTCCTTGAGAGGTACAATG

>gac-let-7a-7

ACACTGTCCTTTGGGGTGAGGTAGTAGGTTGTATAGTTTTAGGGTCACACCCTTCCTGTCAGATAACTATACAACTTACTGTCTTTCCTGAAGTGGCTGTAATGTC

>gac-let-7b-1

TCGTACAGGGTGAGGTAGTAGGTTGTGTGGTTTCAGGGTTGTTATTTTACCCCATCGGGAGTTAACTATACAACCTACTGCCTTTCCTGGAGGG

>gac-let-7b-2

TCGAACAGGGTGAGGTAGTAGGTTGTGTGGTTTCGGGGTAGTGATTTTGCCCGATCAGAAGATAACTATACAACATACTGCCTTCCCTGAAGGG

>gac-let-7c-2

TGTGTGCATCCGGGTTGAGGTAGTAGGTTGTATGGTTTAGAATTACACCCTGGGAGTTAACTGTACAACCTTCTAGCTTTCCTTGGAGTACAC

>gac-let-7d-1

TGTGCTCTGCAGAGTGAGGTAGTTGGTTGTATGGTTTCGCATAATAAACAGCATGGAGATAACTGTACAACCTTCTAGCTTTCCCTGCGGAGTCAC

>gac-let-7d-2

GTGCTCTGCGGTGTGAGGTAGTTGGTTGTATGGTTTCGCATCATAAACAGCCTGGAGATAACTGTACAACCTTCTAGCTTTCCCCGCGGTGTCGC

>gac-let-7e-1

GCTGTCCTTGGGGCTGAGGTAGTAGATTGAATAGTTGTGGGGTTGTCTAACCTCTTTTTGAGATAACTATACAATCTACTGTCTTTCCCAAGGAGACAGC

>gac-let-7e-2

GCTTCCTGCAGAGGCATCTGCCCTTAAGGCTGAGGTAGTAGATTGAATAGTTGTGGGGTCCCTGTCCTCCCTCTGAGATAACTATACAGTCTACTGTCTTTCCTTAGGAGACGG

>gac-let-7f

GTTTGCTCACACAGTATGAGGTAGTAGATTGTATAGTTTTGGGGTAGTGATTTTGCCCTGTTTGGAAGATAACTATACAATCTATTGCCTTCCCTGAGGAGTAGCATCTCTTC

>gac-let-7g-1

ACTTTGGGATGAGGTAGTAGTTTGTATAGTTTTAGGATCACACCAGATCTGGGAGATAACTATACAGCCTACTGTCTTTCTCACGGCAACC

>gac-let-7g-2

CTATGGGATGAGGTAGTAGTTTGTATAGTTTTAGGATCACACCAGATCTGGGAGATAACTATACAGTCTACTGTCTTTCCTATGGTATGCC

>gac-let-7h-1

GGCTGTGCTGTGGTGAGGTAGTAAGTTGTGTTGTTGTTGGGATCATGATTGTGCACCCTGTTCAGGAGATAACTATACAACTTACTGCCTTCCT

>gac-let-7h-2

TAGCTGTGCTGTGGTGAGGTAGTATGTTGTGTTGTTGTGGGGTTTAGGATATTGCAACCCCTAATTTGGAGATACAACTATACAACATACTGCCTTAC

>gac-let-7i-1

GCACTGGCTGAGGTAGTAGTTTGTGCTGTTGGGTCGTGACGCCGCCCGCTATGGAGATGACTGCGCAAGCTACTGCCTTGCCGGTGCTG

>gac-let-7i-2

CCGGTTGAGGTAGTAGTTTGTGCTGTTGGTCGGGTTGTCATATTGCCCCGTTTTGGAGATGACTGCGCAATCTACTGCCTT

>gac-let-7j

GTCTGAGGTAGTTGTTTGTACAGTTTGAGGGTCTGTTATTCTGCCCCAAACAGGAGCTAACTGTACAAGTGACTGCCTTGCC

>gac-mir-1-1

CCTGCTTGGGGAACATACTTCTTTATATGCCCATATGAACACGAGCAACTATGGAATGTAAAGAAGTATGTATCCCAGGTTGGGAAAAA

>gac-mir-1-2

ATTACCTCCTTGGTGCACATACTTCTTTATGTACCCATATGAACATATGATAGCTATGGAATGTAAAGAAGTATGTATTCCTGGTGGGGTG

>gac-mir-100-2

AGCTGCCACAAACCCGTAGATCCGAACTTGTGGTGACTGGCTTCACAAGCTCGTGTCTATAGGTATGTGTCTTC

>gac-mir-101a

TGCCCTGGTTCAGTTATCACAGTGCTGATGCTGTCCCCATCGAAGGTACAGTACTGTGATAACTGAAGGATGGC

>gac-mir-101b

TGAACTGTCCATTTTCAGTTATCATGGTACCGGTGCTGTGTCTGTCTCGAGTACAGTACTATGATAACTGAAGATTGGCAGTGCCATC

>gac-mir-103a

CTCTTTGCGTTCAGCCTCTTTACGGTGCTGCCTTGTGGCGTCTCGATCAAGCAGCATTGTACAGGGCTATGAAGGCTCAGAG

>gac-mir-103b

TGCTCTACGCTTTTAGCCTCTTTACAGTGCTGCCTTGTCTGATCAGGTTCAAGCAGCATTGTACAGGGCTATGACAGCATAGAG

>gac-mir-10544

CAGGGGGGGCTAGGCGTGTCACTGCGTGTCACAGTCACTGCTTGCGCACGGGGCCACGCCCTGCCTACCTGTTC

>gac-mir-10545

CACCGCAGTTTAAGTCTCACACCAGTGCAAAACAAAGATCATGACGCTGCTCAGGTGTGGGACCATGACTGTGGGAGACTCCG

>gac-mir-10b-1

CGTCGTCTATATATACCCTGTAGAACCGAATTTGTGTGATGATACCATAATCACAAATTCGATTCTAGGGGAGTATATGGTCGATGAAAAAACTTC

>gac-mir-10b-2

GTTGTCTATATGTACCCTGTAGAACCGAATTTGTGTGAAATACAAACAGTCGCAAATAGGTCTCTGCAGGAATACATGGGCGAC

>gac-mir-10c

GAGGCGCTGTCTTCTATATCTACCCTGTAGATCCGGATTTGTGTAGAAATCATTAAAATAATCACAAATTCGCTTCTAGGGGAGTATATAGTGGATTTATACACGTCG

>gac-mir-122

ACCGTCAAACGTTGTCCTGCGGAGCTTTGGAGTGTGACAATGGTGTTTGTGTCCTGTGTATCAAACGCCATTATCACACTAAATAGCTACGGTGTGAAATGGGCCTC

>gac-mir-124-3

GGTTGTGTCTCTCCGTGTTCACAGCGGACCTTGATTTAATGTCTTACAATTAAGGCACGCGGTGAATGCCAAGAGAT

>gac-mir-124-4a

GGTTTGAGCTCTTTGTGTTCACAGTGGACCTTGATTTAATTTCAATACAATTAAGGCACGCGGTGAATGCCAAGAGAGAAGCC

>gac-mir-124-4b

CTGGGCTTCCTCTCTCAGTGTTCACAGTGGACCTTGATTTAAAATCATTCAATTAAGGCACGCGGTGAATGCCAAGAGAGAAGCCGCTCTGCTCCAT

>gac-mir-124-6

ACAGGATTGCCACTCCTCGTGTTCACGGCGGACCTTGATTTATTAGCAATACAATTAAGGCACGCGGTGAATGCCAAGAGAGGAATCCCACTATCCACTAAC

>gac-mir-125a-1

GTATGCCCCTCTGTCCCTGAGACCCTTAACCTGTGAGGTCAAAGCAGGTCACAGGTGAGGTCCTTGGGAACAGGGCTGCATTC

>gac-mir-125a-2

CTTCATTGTCCCTGAGACCCTTAACCTGTGATGATATGAAAGGTCACAGGTGAGGTCCTTGGGAAT

>gac-mir-125b-2

GTACCTCTCTCATTCCCTGAGACCCTAACTTGTGACGTTTTGCTGTAATGTGCACGGGTTGGGTTCTTGGGAGCTGCGAGTGGCACTCACACTTC

>gac-mir-125b-3

GTACTTCACCTGCTCCCTGAGACCCTAACTTGTGAGCTCTCTTGATAAAATATCACGGGTTAGGCTCTCGGGACGCGGGTGGACGGCACAATCGACT

>gac-mir-126b

CGGCCCATTATTACTTTTGGTACGCGCTATGCCACTCTCAACTCGTACCGTGAGTAATAATGCA

>gac-mir-128-2

GAGGAGGGGGCCGTTACACTGTCAGAGATGTAGTCTGAGGGTCTCACAGTGAACCGGTCTCTTTTCCTGCTG

>gac-mir-129-1a

GTCCTTTGTGGGTCTTTTTGCGGTCTGGGCTTGCTGTTCCTGAGGAAGTAGCCAGGAAGCCCTTACCCCAAAAAGTATCTGCACTGGAC

>gac-mir-129-1b

TTTCAGGTCTTTTTGCGGTCTGGACTTGCTGTCACATGACAATCCAGGAAGCCCTTACCCCAAAAAGCATCTGCAAGAGGCC

>gac-mir-129-2a

GTCCGTCACGAATCTTTTTGCGGTCTGGGCTTGCTGTGCATACACGACTCTGGAAGCCCTTACCCCAAAAAGCATTGGTGGAGGAC

>gac-mir-129-2b

GTCCTTCACAAATCTTTTTGCGGTCTGGGCTTGCTGTTCATAACTATTAATCTGGGAAGCCCTTACCCCAAAAAGCATTTGCGGAGGGC

>gac-mir-1306

TCCACCACCTCCCCTGCAAACGTCCAGTGACGCAGAGGTAATGGACGTTAGCTCTGGTGGTGATGGATA

>gac-mir-130a

GGCCAGTGCCCCTTTATGTTGTGCTACTGGTGACCCTGCTGGATAAAGCAGTGCAACGTTAAAAGGGCATTGGCCTGAAATGGTCAC

>gac-mir-130b

TGCTCGACACTCTTTCCCTGTTGCACTACTGTGGGAGCTGCAGCAAGCAGTGCAATAATGAAAGGGCATCGGTCAGCTG

>gac-mir-130c-1

GGGGTTTATAGTCTGATGGTGTTTGGTGTTGTTGTCCACTGCCCTTTTCCTCTTGCACTACTGGACATTGAGATGAGCAGTGCAATATTAAAAGGGCATTGGCTGATGGAACAGAGACACACCTGAAACGCCTCC

>gac-mir-130c-2

TTGTCCAAAGCCCTTTTTCTGTTGTACTACTGTGAAATCAGATGAGCAGTGCAATATTAAAAGGGCATTGGCTGACAACACAGCCTCCCCCTCCCCACCACCACC

>gac-mir-132a

GTCTCCATGGTGACCGTGGCATTAGATTGTTACTGTAGCAACAGCACCACTGGTAACAGTCTACAGCCATGGTCGCTAGGGGGCA

>gac-mir-132b

TCCGTCCACCCTGCTGTCTCCATGGCGACCGTGGCTTTAGATTGTTACTCTAGCAACAGCAGCATGGTAACAGTCTACAGCCATGGTCGTTAGGGGCAGGTG

>gac-mir-133a-1

CCACAATGCTTTGCTAAAGCTGGTAAAATGGAACCAAATCACCTCTTGAATGGATTTGGTCCCCTTCAACCAGCTGTAGCTATGCTTTGATG

>gac-mir-133a-2

AATGCTTTGCTAAAGCTGGTAAAATGGAACCAAATCAACTGTTCAATGGATTTGGTCCCCTTCAACCAGCTGTAGCTGTGCATTGA

>gac-mir-133b

CACGCCTTGCCGTGGCTGGTCAAACGGAACCAAGTCAGGTGTTTCTGTGAGGTTTGGTCCCCTTCAACCAGCTACTGCGCCGTG

>gac-mir-135a-1

GCCGTCGTGTCTTATGGCTTTTTATTCCTACGTGATGGTAGATGGGTTCATGTAGGAGTAGAAGCCACTAAACACGCGGTGAG

>gac-mir-135a-2

CACTGTGTACTATGGCTTTTTATTCCTATGTGATGATGAAAGGTATTCGTGTAGGAACAGAAGCCATTTTACACACGGTGAGAGAGCAACACGACAAG

>gac-mir-135b

GCCCAGTGCGCTGTATGGCTTTTTATTCCTATCTGACTGTACTGATGGTTCATATAGGGATGGAAGCCATGCACCGCGCTGG

>gac-mir-135c-1

TGCTGTGTTTTATGGCTTTCTATTCCTATGTGATTTTCTCTGGCATGTCACATAGGGTCTAAAGCCATTGGGTACAGAG

>gac-mir-135c-2

CATTCTCTCCCAAATCAAGCCACATTTGTGTCTTATGGCTTTCTATTCCTATGTGAGTTGGTTTGGCATGTCATGTAGGGTCTAAAGCCATTAGATACTCAGTGG

>gac-mir-137-1a

TCGACCACGGGTATTCTTGGGTTGATAATACAGATGTCGATGTTATTGCTTGAGAATACGCGTAGCTGAG

>gac-mir-137-1b

TCGACCACGGGTATTCTTGGGTTGATAATACAGATGTGGATGTTATTGCTTGAGAATACGCGTAGTCGAG

>gac-mir-137-2a

TCACTGAAAGACTCTCTTCGGTGACGGGTATTCTTGGGTGGATAATACGGATCACGTTGTTATTGCTTAAGAATACGCGTAGTCGAGGAGAGTCCTCTCTTCGCCAA

>gac-mir-1388

TGAGCGCTTGTTTTTCGAGGACTGTCCGACCTGAGAATTGTGATCTCGGGCTCAATCTCAGGTTCGTCAGCCCACGGAAAGCCGACTCGCCTCA

>gac-mir-138a

GCTGCAGCTGGTGTTGTGAATCAGGCCGATGACAGACACCTCCTATAACCCGGCTATTTCACAACACCAGGGTGGCACC

>gac-mir-138ba

GGCGGGGAGGGTCAGCTGGTGTTGTGAATCAGGCCGCTGAGGTGTCGAGACACGGCTTCTTCACAACACCAGGGTTCCTCCTCGC

>gac-mir-138bb

CTGGCCCGGTGGGGCGGGACAGCTGGTGTTGTGAATCAGGCCGCCACAATCAAAGGAACGGCTACTTCCCAACACCAGGGTCTCACCCTCACCCC

>gac-mir-139

TGTATTCTACAGTGCATGTGTCTCCAGTATGTTAGTGATGCTACTGGAGACTCAGCTCTGTTGGAATAACAA

>gac-mir-140

GTCAGTGGTTTTACCCTATGGTAGGTGACATCATGCTGTTCTACCACAGGGTAGAACCACGGACGGG

>gac-mir-142a

GTACAGTGCAGTCATCCATAAAGTAGAAAGCACTACTAAACTCATCGCCACAGTGTAGTGTTTCCTACTTTATGGATGAGTGTACTGTTG

>gac-mir-142b

ACAGTGTTTTCACCCATAAAGTAGAAAGCACTACTAATCTTCAATACACAGTGTAGTGTTTCCTACTTTATGGATGAGTGTACTGT

>gac-mir-143

GCCCATGGTGCAGTGCTGCATCTCTGGTCAGTTGGTAGTCTGAGATGAAGCACTGTAGCTCGGGACGGAGGA

>gac-mir-144

CCCGGACAGGATATCATCTTATACTGTAAGTTTATTAAAGAGACACTACAGTATAGATGATGTACTATCCAGG

>gac-mir-145

TTATTGATTTCCTCTCTCCTGGGGTCCAGTTTTCCCAGGAATCCCTTGACCTATCAGAAAGGGGGATTCCTGGAAATACTGTTCTTGGGGGCGGGGCTTATCCACTCA

>gac-mir-146a

ACTTTGGATGAGTGTTGTACTATGAGAACTGAATTCCATAGATGGTGGCATCTTCAGGTGTCATCTATGGGCTCAGTTCTTTTGGTACGGTACTAATTTGTCAGCTTTAA

>gac-mir-147

ACAGAATCCTTTCTGCACACACTTTGCGTTGACACTCGGTGTGCGGAAATGCTTCTGCTCCACTGGTGGG

>gac-mir-148a

CTTTCCAAATAAAGTTCTGTGAAACACTCCGACTCTTAATTGCTTGCAGTCAGTGCATTACAGAACTTTGTTTTGGGAGT

>gac-mir-150

TCTGGTCACTCCCAATCCTTGTACCAGTGTCGTTACCACGGTGACGCTGGACAGGTTTGGGGGGGGCTGTG

>gac-mir-152a

CTGGTCTAAGTTCTGTGATACACTCTGACTGTGAATCTATGCTAGTCAGTGCATAACAGAACTTTGCCTCGG

>gac-mir-153a

CCAGTGTCATTTTTGTGATGTTGCAGCTAGTAATATGAGCCCAGTTGCATAGTCACAAAAGTGATCATTGG

>gac-mir-153b

CATATCTGTCTGTGTCATTCTTGTGGTTTGCAACTAGTAGTCTGGCTCCAGTTGCATAGTCACAAAAATGATCATGGACTGATGTGGCTGCAGCA

>gac-mir-153c

CATCTCCCAGTGTCATTTTTGTGGTTTGCAGCTAGTACTCTGGCTCCAGTTGCATAGTCACAAAAATGAGCATTGGCAGGTGTGAC

>gac-mir-155

GTTAATGCTAATCGTGATAGGGGTTTTTCTTCATCAGACACCTAACATGTTAGCATTAGC

>gac-mir-15a-2

CTGGTGATGCTGTAGCAGCACGGAATGGTTTGTGGGTTACACTGAGATGCAGGCCATGCTGTGCTGCCACA

>gac-mir-15b-1

CTCAGGGGGCACTCTAGCAGCACATCATGTTTTGCAGATTTCATGCTGAATCGCTCCAAATCATTTTGTGCTGCCACCGTGAGCCCTGGGATTCCCAGTA

>gac-mir-15c

TGTGCCCTTAGACTGCTATAGCAGCGCATCATGGTTTGAAACAATGTGGAAAGGTGCGAACCATTATTTGCTGCTTTAGAATTTTAAGGAAA

>gac-mir-16a

CCTGCCACGCTTTAGCAGCACGTAAATATTGGCGTGTAAGAATAGACCCCAACCCCAATATTAGCAGTGCTGCTTCAGTGTGGCTGG

>gac-mir-16c

TACTGTAGCAGCACGTAAATATTGGAGTTAAGACTTGCTGAAGCCTCCAATATTGATCGTGCTGCTGAAGCAAAGCTGAC

>gac-mir-1788

ACGGTCTCGCTTTCGAGGCTTGTTTTAAGTTGCCTGCGACTCTTATTTGGACACAGGCAGCTAAAGCAAGTCTGGGACGCCGGTGACACACCGAC

>gac-mir-17a-1

GTCTGTGTATTGTCAAAGTGCTTACAGTGCAGGTAGTACTATGCAATACCTACTGCAGTGGAGGCACTTACAGCAATACCCCGAC

>gac-mir-17a-2

GTCATTTGCAGTGTCAAAGTGCTTACAGTGCAGGTAGTTTTATCAAATCTACTGCAGTGAAGGCACTTTCAGCACTATTCTGA

>gac-mir-181a-1

GCTCGCCCCAGTGAACATTCAACGCTGTCGGTGAGTTTCTGCCATGTTGGGAAAACCATCGACCGTTGATTGTACCCTGTGG

>gac-mir-181a-2

GGTGAACATTCAACGCTGTCGGTGAGTTTGAGCTTTGACCAAAACCATCGACCGTTGACTGTACCCTGAGGG

>gac-mir-181a-3

GCTGGCCTCGGTGAACATTCAACGCTGTCGGTGAGTTTTGGTATGGATCACATAGAAACCATCGACCGTTGACTGTGCCCCGAGGCTCGCCTCTCAC

>gac-mir-181a-5

TTTTTCAAGTCTTGGGGAACATTCAACGCTGTCGGTGAGTTTGTGAGCCAGAGAAACCACCGAGTGCTGAGTGTACCCTTACGCTTGAGCTC

>gac-mir-181b-1

CAAAGGTCACAATCAACATTCATTGCTGTCGCTGGGTTGGACTGTGTAGAAAAGCTCACTGAACAATGAGTGCAACTGTGGCCCCGAT

>gac-mir-181b-2

TTAAGGCTGCAGTAAACATTCATTGCTGTCGGTGGGTTTACATGAAAATAGCTCACTGATCAATGAATGCAGACTGCGGTTCAAACA

>gac-mir-181b-3

AAAGGTCACAATCAACATTCATTGCTGTCGGTGGGTTTAACTGTGGAAGAGCTCACTGAACGATGAATGCAACTGTGTCCCAGAT

>gac-mir-181c

AGGTTCCCAGTTCACATTCATTGCTGTCGGTGGGTTGGTGACACGGTCAACTCGCTGGCCGATGGATGGTAACCGTGTCC

>gac-mir-182a

CTCTCTGGTGGTGTTTGGCAATGGTAGAACTCACACTGGTGAGGTAGCTGGATCCGGTGGTTCTAGACTTGCCAACTACTGCCCGAGAGCGTCCGGCAGC

>gac-mir-182b

CTCTCCCACAGTGTTTGGCAATGGTAGAACTCACTCTGGTGGGCTGGAAGGATCCGGTGGTTCTAGACTTGCCAACAACTGACCGAGAGCT

>gac-mir-183a

CTGTGTATGGCACTGGTAGAATTCACTGTGAGAGCTCACTATCAGTGAATTACCATAGGGCCATAAACAG

>gac-mir-183b

CTCCTCCTGTTCTGTGTATGGCACTGGTAGAATTCACTGTCGCAGAGAACACTATCAGTGAATCACCATAGGGCCATAAACAGAGTAGAGACAGAACCACA

>gac-mir-184a

GTCGAGCACATCTCCTTATCACTTTTCCAGCCCAGCTATCTATCAAATGTTTGTTGGACGGAGAACTGATAAGGGTATGTGTCTGA

>gac-mir-184b

GTCGCCCACATCTCCTTATCACTTTTCCAGCCCAGCTATAGATTATGTTTCCGTTGGACGGAGAACTGATAAGGGCATGTGCATGA

>gac-mir-18a

TTGTGCTAAGGTGCATCTAGTGCAGATAGTGAAATAGACTAGCACCTACTGCCCTAAGTGCTCCTTCTGGCATAAG

>gac-mir-18b

TAGTGCTTATGCTAAGGTGCATCTAGTGCAGATAGTGAAGTAGACTAGCACCTACTGCCCTAAGTGCCCCTTCTGGCATAAGGAGCT

>gac-mir-18c

GTCTTCCAGCTAAGGTGCATCTAGTGTAGTCAGTGAAGTAGCTCAGTATCTACTGCCCTAGTTGCTCCTTCTGGCTGGAGGGCT

>gac-mir-1905-1

GTGCAGGTCTGCTGCTGGATGCGTTTGATGGTGGAGCCCTTGGGCCCCACCACCAGCCCCACGACGCGGTATGGCACTCTCA

>gac-mir-1905-2

GTGTGGGTCTGCTCCTGGATGCGCTTGATGGTGGCACCCTTGGGTCCTACAACAAGCCCCACCACGCGGTAAGGCACCCGCA

>gac-mir-190a-1

ACCGGAGGCCTTCTCTGCAGGGCTCTGTCTGATATGTTTGATATATTAGGTTGTTATTTAGTTCCAACTATATATCAAACATATTCCTACAGTGTCCCGCCCTGTCTACAG

>gac-mir-190a-2

TGGTGTTTACCTGGAAGCCTTCTCTGCAGGGCTCTGTCTGATATGTTTGATATATTAGGTTGTTAATAAGTCCAACTATATATCAAACATATTCCTACAGTGTCCCGCCCTGTCTCCAG

>gac-mir-190b

GCTAGAAGCTGTGTGATATGTTTGATATTCGGTTGTTCTTGTGCTTCGTCGTGTCAACTAAATATCAGACATATTCCTACAGAGTCTGGCA

>gac-mir-191

GGATTGTAACGGAACCCATAATGCAGCTGTGATTCTTCGCTCCAGCTGGTTACGGGGTCCGTTTCTATCCCACAGCGCGGAGGTCTT

>gac-mir-192

CACAAAGCGATGACCTATTAATTGACAGCCAGTGGTTGTAAACTCTGCCTGTCAGTTCTCTAGGCCACTGCTGTGTTTCTCCC

>gac-mir-193a-2

GTGTGTTAGAGGCTGGGTCTTTGCGGGCAAGGTGAGTCCTACATTTGTTCAACTGGCCTACAAAGTCCCAGTTTCTGGCTCATGTGACCACC

>gac-mir-194a

CGCTGGATGTAACAGCAACTCCATGTGGAAGCTGCGTCCAGTTCCAGTGGAGGTGCTGTTACCTGCAGAGGACCACC

>gac-mir-194b

TGGGTCTCACCAGCTGTAACAGCATCTCCATATGGAATAATTCTTGCTTCCAGTGGAGCTGCTGTTATCTGTGGTGGG

>gac-mir-196a-1

AGCTGAAGCGTGGTTTAGGTAGTTTCATGTTGTTGGGGTTGGCTTCCTGGCTCGGCAACAAGAAACTGCCTTGATTACGTCAGTTCGTCTTCATCAAGGGC

>gac-mir-196a-2

CGGGTGGTTTAGGTAGTTTCATGTTGTTGGGGTCCATTTCTAACTCTGCAACACGAAACTGTCTTAACTGCCCCA

>gac-mir-196b

TGTGTGATTTAGGTAGTTTCAAGTTGTTTGGCTGGATGCTTAAGATCACAGGGACCTGAAACGGCCTTAATCACACCA

>gac-mir-196d

GCGGGCTGTTGCGTGGTTTAGGTAGTCTCATGTTGTTGGGCTATTATATTTCTCCCACAACACGAAACTGCCTTGATTACCTCAGT

>gac-mir-199-1a

CCGCCTGCCCAGTGTTCAGACTACCTGTTCAGGAAGTAGTGGTTGTACAGTAGTCTGCACATTGGTTAGGCTG

>gac-mir-199-2a

CCCCCCCCTGCCTGCCCAGTGTTCAGACTACCTGTTCATCCGGCTACAGCTGAACAGTAGTCTGCACATTGGTTAGGCTGGGCTGGGACACGCACAC

>gac-mir-199-3a

CCGGCTCCGTCCACCCAGTGTTCAGACTACCTGTTCATTATCATACTGGTGTACAGTAGTCTGCACATTGGTTAGACTGGGCATGG

>gac-mir-19a-1

GCAGTTCTCTGCTAGTTTTGCATAGTTGCACTACAAGAATAGATGAGTTGTGCAAATCTATGCAAAACTGATGGTGGCCTG

>gac-mir-19a-2

GCAGTTCTCCGTTAGTTTTGCATAGTTGCGCTCCAAGAAGAAATGCGTTGTGCAAATCTATGCAAAGCTAACGGTGGCCTGCGTTTCC

>gac-mir-19b-1

GGTCAGTTTTGCTGGTTTGCATCCAGCTTTAACTATTGTTTGCTGTGCAAATCCATGCAAAACTGATT

>gac-mir-19b-2

TGGGTCTCTGGTTAGTTTTGCTGGTTTGCTTTCAGCTTTTCTCTGTAGTGCTGTGCAAATCCATGCAAAACTGAT

>gac-mir-19c

GGAGTTTTGTTGGTTTGCTTTCAGCCTCTGCCAGTCTCTGCTGTGCAAATCCATGCAAAGCTCT

>gac-mir-200a

TCCCAGGATCCATCTTACCCGACAGTGCTGGATTGTACGTCTGTTGTTCTAACACTGTCTGGTAACGATGTTTCCTGGGTGGC

>gac-mir-200b

GGTGATTATCTCCATCTTACGAGGCAGCATTGGATATTATCTCTCTCTCTAATACTGCCTGGTAATGATGATGATAG

>gac-mir-202

TCCTTTTTCCTATGCATATACCTTTTTCAGATGTAACTTTAAAGAGGTATAGGGCATGGGAAAATGGGG

>gac-mir-204-1

ATGTGACTTATGGCCTTCCCTTTGTCATCCTATGCCTGGACTCATACAAAAGGGGCTGGGAAGGCAAAGGGACGCCCAGTCGTCATAC

>gac-mir-204-2a

TGTGACCCGTGGGTTTCCCTTTGTCATCCTTTGCCTGGAGGTGTCTGTAAGGCAGCGGCAGCAAAGGGAGGCTCAGCCATCACTCACT

>gac-mir-204-2b

TGGGACCTATGGGCTTCCCTTTGTCATCCTATGCCTGGAGCTCGGATAAGGCAGGGACAGCAAAGGGATGCTCAACTGTCACCACCGACTTCA

>gac-mir-205-1a

TGTGTTCTGTCCTTCATTCCACCGGAGTCTGTCTGTTTCACACCAGATTTCAGTGGTGTGAAGTGTAAGAGACATGG

>gac-mir-205-1b

TGTATTCTATCCTTCATTCCACCGGAGTCTGTGTAAAGTGCCAGTCAGATTTCAGTGGTTTGAAGAGTAAAACGCATGGA

>gac-mir-206-1

TGTTGCCCCTCGCGAGGACATGCTTCCTTATATCCCCATATCAATACACCACTTATGGAATGTAAGGAAGTGTGTGGTCTCAAGGGG

>gac-mir-20a-1

GTTTCAGCAGTACTAAAGTGCTTATAGTGCAGGTAGTGTTTTTCTCCTATCTACTGCAATATAAGCACTTGAAGTACTTCTAACTTGCTGCATCTTTCCGCAAGCTTT

>gac-mir-20a-2

GTGTGTCAGTAGTATTAAAGTGCTTATAGTGCAGGTAGTTCTTTAAAATTCTACTGCAGTGTGAGCACTTGAAATACTTCTAGAT

>gac-mir-20b

TCGACTTTGCTGTTCCAAAGTGCTCACAGTGCAGGTAGGGCACACGGATCTACTGCAGTTTGTGCACTTCAGGTATTGCCGGTCACCTTC

>gac-mir-210

TCTAAAAGCAGGTAAGCCACTGACTAACGCACATTGTGCCAGTGTCCAAATTCACTGTGCGTGTGACAGCGGCTAACCTGGTTTTGGGAGCACTTCT

>gac-mir-212a

GTCAGTGCATCAATACCTTGGCTCTAGACTGCTTACTGCTAAAACTGTCCCGAAGTACAGTAACAGTCTACAGTCATGGCTACTGAAGTCTGGC

>gac-mir-212b

GGCCAGAGCATCAGCACCTTGGCTTTAGACTGCTTACTGCTTATACAGTGTCACAGTACAGTAACAGTCTACAGTCATGGCTACTGAAGCCTGGC

>gac-mir-214a

TGGCTGAAGGAGTTGCAGTGTGTCTGCCTATCTACACTTGCTGTGCAGAATAACCTCCAACCTGTACAGCAGGCACAGACAGGCAGATAGACGTCAGCCTGCC

>gac-mir-216a-1

TTGGTGAAATCTCAGCTGGCAACTGTGAGTCGTTCACTAGCTGCTCTCACAATGGCCTCTGGGATTATGCTAA

>gac-mir-216a-2

GCTGTGATCCAGTTTAATCTCAGCTGGCAACTGTGAGCTTTGATCGCCTCACAGAGCCAACTAAGATTCACCTGAACGCAACACCATCT

>gac-mir-216b

TGACTGGGTAATCTCTGCAGGCAACTGTGATGGTGCTTTAAATTCTCACAATGACCTGGAGAGATTCTGCAGTT

>gac-mir-217a

AGGAAAAACTCTCCGATGCGGATGATACTGCATCAGGAACTGATTGGCTGATGCTCAAATGTCAACAGTACCTGATGCATTGCCTTCAGCATCGCAAGA

>gac-mir-217b

CTGATGTTGGTGATACTGCATCAGGAACTGAATGGAGACCCATGAGAGGCCATCGGTTCCTGATGCACGGTCCTCAGCATCTGAAGA

>gac-mir-2184

CCTCCGCCCTGAACAGTAAGAGTTTATGTGCTGTTGTTCATCTGTCAGCACATGAGCTTTTACGGTTAAGGGAGGAAGAG

>gac-mir-2187a

TGTGAATTGATGATTCTGGCTTTAATTAGTATAGCCTGTTTTAGTGATATCAGCAATTCTTTACAGGCTATGCTAATCTGTGCCAGAATCAGCAATGCAATTGG

>gac-mir-2187b

GATCTGATGGTTCTGGCTTTAATTAGTACAGCCTGTATTAGTAATGTCATTTATTCTTTACAGGCTATGCTAATCTATGCCAGAATCAGCAATGCAATGG

>gac-mir-2188

GAATAATATCAGTGGTGTGTGAAAGGTCCAACCTCACATGTCCTGTGCGGCTGAAGGAAGGCTGTGTGAGGTCAGACCTATCCCACACGGCTCGTATTCTTCCC

>gac-mir-218a-2

GGGTGTTCCTTTGTGCTTGATCTAACCATGTGTCTGAACGGTTCCTATAGTAAAACATGGTTCTGTCAAGCACCATGGAACGGC

>gac-mir-218b

CAGGACGCCATTGTGCTTGATCTAACCATGCAGTGCATCGTCTGTCCATGGTTGTGCCAAGCACTTTGGAGGCTTG

>gac-mir-219-1

CCGAGTCTCTAGCGACTGATTGTCCAAACGCAATTCTTGAGAAAACTCCAAATTCAACCCCCAAGAATTGTGTATGGACATCTGTTGCTGCAGACTC

>gac-mir-219-2

TCTGAGCTCTAGCTGCTGATTGTCCAAACGCAATTCTTGTAAAATCTCAAATGCAAACCCGAGAATTGTGCCTGGACATCTGTCGCTGGTCGCTCC

>gac-mir-219-3

CGGAGCTGATTGTCCAAACGCAATTCTTGCGTCTGCCTTTGTGAAACCAGGAGTTGTGGATGGACATCACGCCCCTGAC

>gac-mir-219-4

GTTTGACAGGGATTCTGGTGTTGATTGTCCAAACGCAATTCTTGTGTTACTTCTCTATATCCAGGAGTTGTGGATGGACATCATGCCCCCGACTCTCATTCACTTC

>gac-mir-2196

GGGTAGTTGCTGCGGATGTTCCTCTCTGTGCTCCCGTTTGGAACCGTCCCGAAGCGAAAAGGAGGGTAATGCTCATGCGGTTTTTGGAACTG

>gac-mir-21a

TCTGTCACTCTCTCGGCCTGTCCGATAGCTTATCAGACTGGTGTTGGCTGTTAAGATTGCAAGGCGACAACAGTCTGTAGGCTGTCTGACATTTCGGGCTTTCATCT

>gac-mir-21b

CTCTCCCGTCCCGTCAGCTAGCTTATCAGACTGGTGTTGGCTGTTCAATCACCACGGCAACAGCGGTCTGTAAGCTGGCCGAAGTTATGGGCCTTCT

>gac-mir-221a

GTCCTGAACCTGGCATACAATGTAGATTTCTGTGTGGTTAAATTCAACAGCTACATTGTCTGCTGGGTTTCTGGCCAGTAACATAA

>gac-mir-221b

GTTCTGGACCTGGCATTCAGTGTAGGATTCTGTGTGTGTCAATCTACAGCTACATTGTCTGCTGGGTTTCAGGCCCTCA

>gac-mir-222a

CGGTTGCTCAGTAGGCAGTGTAGATCCTGTGTAGCAATCAGCAGCTACATCTGGCTACTGGGTCTCCG

>gac-mir-222c

GCCGTGCATCCTCGGGTCGGCCCCGCGGGTGCTCAGTAGTCAGTGTAGATCCTGTGCGGCTGGCAGCAGCTACATCTGGCTACTGGGTCTCTGCCGGCA

>gac-mir-223

CAGGCCCCCCACTTAGTGTATTTGACAAGCTGAGTTGGACACTCTGTATCTCTGAGTGTCAGTTTGTCAAATACCCCAAGTGAGG

>gac-mir-22a-1

GCTGACCTACAGCAGTTCTTCACTGGCAAGCTTTATGTCCTCATCTACCAGCTAAAGCTGCCAGCTGAAGAACTGTTGTGGTCGGC

>gac-mir-22a-2

GCTGACGCACGGCAGTTCTTCACTGGCAAGCTTTATGTTCCCGTGCACATGCTAAAGCTGCCAGCTGAAGAACTGCTGTGGTCAGC

>gac-mir-22b

GTTGCCTCACAGTCGTTCTTCACTGGCTAGCTTTATGTCCCACGCCCCGCGCTAAAGCTGCCAGTTGAAGAGCTGTTGTGTGTAAC

>gac-mir-23a-1

CACGGCTGTGGTGGGGAGGGTTCCTGGCACCGTGATTTGATGCACAAAGAAAAACAAAATCACATTGCCAGGGATTTCCACTCCTTCACGGCACCG

>gac-mir-23a-2

TCTGATGGCCAGGGGAATTCCTGGCAGAGTGATTTTTGAGACTACCGCACTGAATCACATTGCCAGGGATTTCCAATGGCT

>gac-mir-23a-3

AGCTGGAAGGATTCCTGGCAGAGTGATTTTGTTGTGATGTAATGTAAATCACATTGCCAGGGATTTCCAACCAGCT

>gac-mir-23b-2

GGCTGTGAGGGTTCCTGGCGTGCTGATTTGTGACTTATGATAAAATCACATTGCCAGGGATTACCACACAGCC

>gac-mir-24-2

GGGTCAGTCTCCTGTGCCTGCTGTGCTGATAATCAGTGTGTGACGTTGGCTGGCTCAGTTCAGCAGGAACAGAGGACCGGTC

>gac-mir-24-3

GGAGAGCGAGCAGAGATATTCAGGGTTGGCTTCCTGTGCCTACTGAGCTGATAATCAGTCCTATACAACCACTGGCTCAGTTCAGCAGGAACCGGAGTCCAGTCCACTCCACACCGGACCAGAAGAC

>gac-mir-24-4a

CCTCCCGTGCCTACTGAACTGGTATCAGTGTTTTTTCTAAAAACTGGCTCAGTTCAGCAGGAACAGGAGT

>gac-mir-24-4b

GAGTTGTGCACTCCTGTGCCTACTGAACTGGATTCAGTGTGTCTTTGCAAGAACTGGCTCAGTTCAGCAGGAACAAGAGT

>gac-mir-24-6

TACCTGGGCTCAACCTCCTGTGCCTACTGAGCTGATAACAGTTAGATGTTAAACAAACACTGGCTCAGTTCAGCAGGAACCGGAGTTAGGCCCTCTAGAAAC

>gac-mir-26a-1a

TGGGTCTGTTTCAAGTAATCCAGGATAGGCTTGTTAAAGTGGGAAAAGCCTATTTGGGATGACTTGGTTCAGAAA

>gac-mir-26a-1b

TGGGTCTGGGCCTGGTTCAAGTAATCCAGGATAGGTTTGTTCAGACAAGCACAGCCTATTCCGGATGACTTGGTTCAGGAACGCTAC

>gac-mir-26a-2

AGAGACTGGCTGTGATCTGGTTCAAGTAATCCAGGATAGGCTGTGTGTATGTTTGTGAGCCTACTCGTGATTACTTGCACTGGGCTGCAGCCGCTGGGCAA

>gac-mir-26a-3

GCCAGACTGTCGCTGTGACCGGGTTCAAGTAATCCAGGATAGGCTTTCTGCGTCTGTGTTGGCCTGTGCTTGATTACTTGCACTCGGGGTCAGCAACTAACC

>gac-mir-26b

GGCCTCTGCCTGGTTCAAGTAATCCAGGATAGGCTGGTTAACACTGGCACGGCCTATTCTTGATTACTTGTTTCAGGAACAGGCCATTAGC

>gac-mir-27a

AGTTTGACGGAGGGGCAGGACTTAGCTCGCTCCGTGAACAGTCCAGTGAAAGCCTGTGTTCACAGTGGCTAAGTTCCGCTCCTCACAAGCCC

>gac-mir-27b

AGGCACAGAGCTTAGCTGATTGGTGAACAGTGATTGATTTCCTCTTTGTTCACAGTGGCTAAGTTCTGCACCTG

>gac-mir-27c-1

GGCTGTGCGGCAGCAGGACTTAACCCACATGTGAGCAGTGAGTGTCTGCCATGTTCACAGTGGTTAAGTTCTGCCGCC

>gac-mir-27e-1

CACTTGCTGAAGGCACAGAGCTTAGCTAATTGGTGAGCAGTGATCCCTGCTATGTGTTGTTCACAGTGGCTAAGTTCAGTGCCTGAGGTGAAATAGGGGA

>gac-mir-27e-2

TTTCTGAAGATGCAGAATTTAGCTCATTAGTGAGCATTGAACCAAACAGGAATTGTTCACAGTGGCTAAGTTCAGTGTCTGACATGAAAGAAT

>gac-mir-2985-1a

AAGGATCCTCATTAAGGTGGGTGGAATAGTATAACAATGTGTCCAATGTTGTTATAGTATCCCACCTACCCTGATGTAGCT

>gac-mir-2985-2a

AGGGTTCACAGTTAAGGTGGGTGGAGTAGTGTAACAATATGTTCTACGTGTTGTTATGGTATTCCACCTTCCCTGGTGTGCC

>gac-mir-2985-2b

GTTAAGGTGGGTGGAATAGTGTGACAATAGGAGACAGTAACAAAGCTGATACTGTCCTATTGCTGCGATATTCCACCTACCCTGCTGTGCCT

>gac-mir-2985a

AAGGCTCAGCATTAAGGTGGGTGGAATAATATAACAATATCCTCCATGTTGTTATAGTACTCCACCTACCCTGATGT

>gac-mir-2985b

TCTGCATTAGGGTGGGTGGGATAATATAACAATATCTTCCATGTTGTTATAGTATTCCACCCACCCTGATGTTT

>gac-mir-29a-1

TTTTTCTCCTCTAAAAGATAACCGATTTCTTCTGGTGCATAGAGCCCGCTGCAGCCTTCTAGCACCATTTGAAATCGGTTATGAAACTGTGGATCAATGC

>gac-mir-29a-3a

AAGTTGACCGATTACTTTTGGTGTTCAGAGTCTGCTTTTGTTTCTAGCACCATTTGAAATCGGTTACAGTGA

>gac-mir-29a-3b

GGAACTGGTTTCAGATGGTGTCTTAGAGTACAAACCCCTGTCTAGCACCATTTGAAATCGGTGGTCTTGGG

>gac-mir-29b-1a-1

CCCTTGGAAGCTGGTTTCAGATGGTGGCTTAGAGTGTTGGCATCTTTCTAGCACCATTTGAAATCAGTGTTCTCGGGG

>gac-mir-29b-1a-2

CCCTTGGAAGCTGGTTTCAGATGGTGGCTTAGAGTGTTGGCATCTATCTAGCACCATTTGAAATCAGTGTTCTTGGGG

>gac-mir-29b-1b

TCTCGCACAGGCTGACCGCTTTCGTGTGGTGTTCAGAGTTCCCAACACGTCTAGCACCATTCGAAATCGGTTACTATGTGCAGGGACTTCCAGCAG

>gac-mir-29b-2a

TCTTCCCCTAGAAGCTGGTTTCATATGGTGGTTTAGATGTGTGTTCCCATTGTCTAGCACCATTTGAAATCAGTGTTCTTGAGGA

>gac-mir-29b-2b

CGGAAACTGATTTCATTGGGTGAGGTAGATGTTTGTACCCCGTCTCTAGCACCATATGAAATCAGTGTTCCTGG

>gac-mir-301a

GCTGTTGGCGGGTGCTCTGACTTCATTGCACTACTGTTTGAACACAGCTAGCAGTGCAATAGTATTGTCAAAGCATCTGAAAACTGC

>gac-mir-301b

GAGGTCAGCTGCTTTGACGATGTTGCACTACTGTACCATCCGCTAAAGCAGTGCAATAGTATTGTCATAGCATTCGGCCTTT

>gac-mir-301c

CAGCTGCTTTGACAATGTTGCACTACTGTACCATCCATTCTAGCAGTGCAATAGTATTGTCATAGCAT

>gac-mir-30a

TGAACAAGGTAGTTTGGGGCTGTAAACATCCCCGACTGGAAGCTGTCCATCTGCAGAGCTTTCAGTCCGATGTTTGCTGCTGCCAACTGCCTCCAGCT

>gac-mir-30bb

TTCTGACTCAGTTCATGTAAACATCCTACACTCAGCTGTTTTCAATTGCAGCAGAGGCTGAGAGAAGGTTGTTTACTTGAACCGGCTGGA

>gac-mir-30c-1

TCCAGGAAGTGTAAACATCCTACACTCTCGGTGTGGCGCCCCTGGTGGCCGGGAGTGGGAATGTTTATACTGCCTGGCT

>gac-mir-30c-2

CAGGCATGTAAACATCCTACACTCTCAGCTGTGTTGTCATGGAGCTGGGAGAAGGGTGTTTACTCTTCTGGTGATGGAGAAACCTCATCAAGACGAA

>gac-mir-30e-1

CGGCAACCTGGAGGTGCTGTAAACATCCTTGACTGGAAGCTGTGGTTTTGGACGTCAAGGGCTTTCAGTCGGATGTTTGCAGCATCTTATTGCCTC

>gac-mir-30e-2

CAGGCTACTGTAAACATCCTTGACTGGAAGCTGGTTTTAGGTTCTGGGGCTTTCAGTCGGATGTTTGCAGCAGCCAACT

>gac-mir-3120

TGGCTCATGGGGCAGGCTGACGTCTATCTGCCTGTCTGTGCCTGCTGTACAGGTTGGAGGTTATTCTGCACAGCAAGTGTAGATAGGCAGACACACTGCAACTCCTTCAGCCAGTTGGTGTTT

>gac-mir-31a

AGGCAAGATGTTGGCATAGCTGTTAAATTGAAATCCCTGCTATATCAACATATTGCCA

>gac-mir-338-1a

GGTTGCTTCATGGAACAATATCCTGGTGCTGCCTGAGTAATCCACTTAAAGACTCCAGCATCAGTGATTTTGTTGCAGGCGGTGACC

>gac-mir-338-1b

GGTCTTTCCCTGCAACAACATCCTGGTGCTGCCTGAGTAATTGTCAAAAACTCCAGCATCAGTGATTTTGTTGCGGGGGG

>gac-mir-338-2

TCTCTGTTGCCTCCTGGAAACAACATCCTGATGCTGTCTGGGTGTGAAAAGCAGAACTCCAGCATCAGTGATTTTGTTGCCAAGGGGACGACTTT

>gac-mir-33a

CTGTGCGGGTGCCAGGAGGTGGCTGTGGTGCATTGTAGTTGCATTGCATGGTCCTTAAAGCCGAGTGCAATGTGTCTGCAGTGCAGTACAGAGGCCTCCCCAATCTGCA

>gac-mir-33ba

CGGCCAGAGCTGTGGTGCATTGTAGTTGCATTGCATGTGTGTCTCAGCGGAGTGCAATGCACCTGCAGTGCAACACGGAGCTGGGCCACG

>gac-mir-33bb

CAGCGCTGAGGAGCTGTGGTGCATTGTAGTTGCATTGCATGTTGTCTGACGCTGCAATGGATCTCCTCTGCAACACAGAACTCAGTC

>gac-mir-34a

CTGCTGTGGGTGTTTCTCTGGCAGTGTCTTAGCTGGTTGTTGTGAGGAGTGAGAACGAAGCAATCAGCAAGTATACTGCCGCAGAAACTCTTCGC

>gac-mir-3618

ATATTAAGCTGAATGCATTGTGATTTCCAATAATTGAGACAGTGATTCTGAAAGCTGTCTACATTAATGAAAAGAACAATGTAGTCAGCTTAGCAT

>gac-mir-365-2b

AGGCAGCAAGAAAAGTGAGGGACTTTTAGGGGCAGCTGTGTTTCATTAACCCAGTCATAATGCCCCTAAAAATCCTTATTGCTCTTGCAATGGTCAGC

>gac-mir-375a

GTATTTGCTTCACGTTGAGCCACACGTACAATACCTGAAGATGAAGTTTTGTTCGTTCGGCTCGCGTTACGCAGGT

>gac-mir-425

GGTGTTTTTCCTCAATGACACGTTTTCTCCCGGATTGCTGACAACAGTGAACGCAACCGGGAACTTCGTGTCAGCCAAGAATGACACC

>gac-mir-429a

AGCCTGTTGATTGGCGTCTTACCAGACATGGTTAGATGTAATTAATGGTGTCTAATACTGTCTGGTAATGCCGTCCATTAAATGGCA

>gac-mir-430a-1

TGGTCTTCATGGTTACCCTTACAAAAACACTGACTTTGGTTCTCAATACAGTAAGTGCTTTTTGTTGGGGTAGTTTTTGCTGA

>gac-mir-430a-10

AACACTCTCAGATAACTTCAAATAGAGCCACTGGTGATGATTTGGTTCATAAGTGCTTCTCTTTGGGGTTGTCTTA

>gac-mir-430a-11

GTCTTCCAGATTTGGCCATTAGGATTACCCTTACAAAAACACTGACTTTGGTTCTCAATACAGTAAGTGCTTTTTGTTGGGGTAGTTTTGCTGATCTCTGCAAC

>gac-mir-430a-12

CAATATTCTCCATTGTGTCCTGATTTGGTCTTCATGGTTACCCTTACAAAAACACTGACTTTGGTTCTCAATACAGTAAGTGCTTTTTGTTGGGGTAGTTTTGCTGATCTCTGCAACA

>gac-mir-430a-13

TGGTCTTCATGGTTACCCTTACAAAAACACTGACTTTGGTTCTTAATACAGTAAGTGCTTTTTGTTGGGGTAGTTTTGCTGATCTCTGCAACA

>gac-mir-430a-14

TGGTCTTCATGGTTACCCTTACAAAAACACTGACTTTGGTTCTCAATACAGTAAGTGCTTTTTGTTGGGGTAGTTTT

>gac-mir-430a-15

TCAATGGAATCCAATTCAGATAACCTCAAACTGAGACACTGATGATTCTTCAGTTCATAAGTGCTTCTCTTTGGGGTTGTCTT

>gac-mir-430a-16

AACACTCTCAGATAACTTCAAATAGAGCCACTGGTGATGATTTGGTTCATAAGTGCTTCTCTTTGGGGTTGTCTTA

>gac-mir-430a-17

TGGTCTTCATGGTTACCCTTACAAAAACACTGACTTTGGTTCTCAATACAGTAAGTGCTTTTTGTTGGGGTAGTTTT

>gac-mir-430a-18

TCAATGGAATCCAATTCAGATAACCTCAAACTGAGACACTGATGATTCTTCAGTTCATAAGTGCTTCTCTTTGGGGTTGTCTT

>gac-mir-430a-19

AACACTCTCAGATAACTTCAAATAGAGCCACTGGTGATGATTTGGTTCATAAGTGCTTCTCTTTGGGGTTGTCTTA

>gac-mir-430a-2

TCAATGGAATCCAATTCAGATAACCTCAAACTGAGACACTGATGATTCTTCAGTTCATAAGTGCTTCTCTTTGGGGTTGTCTT

>gac-mir-430a-20

TTCTGTGTGTCCAGATTTGGCCATTAGGATTACCCTTACAAAAACACTGACTTTGGTTCTCAATACAGTAAGTGCTTTTTGTTGGGGTAGTTTTGCTGATCTCTGCAAC

>gac-mir-430a-21

TGGTCTTCATGGTTACCCTTACAAAAACACTGACTTTGGTTCTCAATACAGTAAGTGCTTTTTGTTGGGGTAGTTTT

>gac-mir-430a-22

TCAATGGAATCCAATTCAGATAACCTCAAACTGAGACACTGATGATTCTTCAGTTCATAAGTGCTTCTCTTTGGGGTTGTCTT

>gac-mir-430a-23

AACACTCTCAGATAACTTCAAATAGAGCCACTGGTGATGATTTGGTTCATAAGTGCTTCTCTTTGGGGTTGTCTTA

>gac-mir-430a-24

TGGTCTTCATGGTTACCCTTACAAAAACACTGACTTTGGTTCTCAATACAGTAAGTGCTTTTTGTTGGGGTAGTTTT

>gac-mir-430a-25

TCAATGGAATCCAATTCAGATAACCTCAAACTGAGACACTGATGATTCTTCAGTTCATAAGTGCTTCTCTTTGGGGTTGTCTT

>gac-mir-430a-26

AACACTCTCAGATAACTTCAAATAGAGCCACTGGTGATGATTTGGTTCATAAGTGCTTCTCTTTGGGGTTGTCTTA

>gac-mir-430a-27

TTTCTCCTCCAGATTTGGCCATTAGGATTACCCTTACAAAAACACTGACTTTGGTTCTCAATACAGTAAGTGCTTTTTGTTGGGGTAGTTTTGCTGATCTCTGCAACA

>gac-mir-430a-28

TGGTCTTCATGGTTACCCTTACAAAAACACTGACTTTGGTTCTCAATACAGTAAGTGCTTTTTGTTGGGGTAGTTTT

>gac-mir-430a-29

TCAATGGAATCCAATTCAGATAACCTCAAACTGAGACACTGATGATTCTTCAGTTCATAAGTGCTTCTCTTTGGGGTTGTCTT

>gac-mir-430a-3

AACACTCTCAGATAACTTCAAATAGAGCCACTGGTGATGATTTGGTTCATAAGTGCTTCTCTTTGGGGTTGTCTTA

>gac-mir-430a-30

AACACTCTCAGATAACTTCAAATAGAGCCACTGGTGATGATTTGGTTCATAAGTGCTTCTCTTTGGGGTTGTCTTA

>gac-mir-430a-31

TTCTGTGTGTCCAGATTTGGCCATTAGGATTACCCTTACAAAAACACTGACTTTGGTTCTCAATACAGTAAGTGCTTTTTGTTGGGGTAGTTTTGCTGACCTCTGCAAC

>gac-mir-430a-32

TGGTCTTCATGGTTACCCTTACAAAAACACTGACTTTGGTTCTCAATACAGTAAGTGCTTTTTGTTGGGGTAGTTTT

>gac-mir-430a-33

TCAATGGAATCCAATTCAGATAACCTCAAACTGAGACACTGATGATTCTTCAGTTCATAAGTGCTTCTCTTTGGGGTTGTCTT

>gac-mir-430a-34

AACACTCTCAGATAACTTCAAATAGAGCCACTGGTGATGATTTGGTTCATAAGTGCTTCTCTTTGGGGTTGTCTTA

>gac-mir-430a-35

TTTCTCCTCCAGATTTGGCCATTAGGATTACCCTTACAAAAACACTGACTTTGGTTCTCAATACAGTAAGTGCTTTTTGTTGGGGTAGTTTTGCTGATCTCTGCAAC

>gac-mir-430a-36

TGGTCTTCATGGTTACCCTTACAAAAACACTGACTTTGGTTCTCAATACAGTAAGTGCTTTTTGTTGGGGTAGTTTT

>gac-mir-430a-37

TCAATGGAATCCAATTCAGATAACCTCAAACTGAGACACTGATGATTCTTCAGTTCATAAGTGCTTCTCTTTGGGGTTGTCTT

>gac-mir-430a-38

AACACTCTCAGATAACTTCAAATAGAGCCACTGGTGATGATTTGGTTCATAAGTGCTTCTCTTTGGGGTTGTCTTA

>gac-mir-430a-39

TCTCCAGATTTGGCCATTAGGATTACCCTTACAAAAACACTGACTTTGGTTCTCAATACAGTAAGTGCTTTTTGTTGGGGTAGTTTTGCTGATCTCTGCAAC

>gac-mir-430a-4

TTTCTCCTCCAGATTTGGCCATTAGGATTACCCTTACAAAAACACTGACTTTGGTTCTCAATACAGTAAGTGCTTTTTGTTGGGGTAGTTTTGCTGATCTCTGCAAC

>gac-mir-430a-40

TGGTCTTCATGGTTACCCTTACAAAAACACTGACTTTGGTTCTCAATACAGTAAGTGCTTTTTGTTGGGGTAGTTTT

>gac-mir-430a-41

TCAATGGAATCCAATTCAGATAACCTCAAACTGAGACACTGATGATTCTTCAGTTCATAAGTGCTTCTCTTTGGGGTTGTCTT

>gac-mir-430a-42

AACACTCTCAGATAACTTCAAATAGAGCCACTGGTGATGATTTGGTTCATAAGTGCTTCTCTTTGGGGTTGTCTTA

>gac-mir-430a-43

TTTCTCCTCCAGATTTGGCCATTAGGATTACCCTTACAAAAACACTGACTTTGGTTCTCAATACAGTAAGTGCTTTTTGTTGGGGTAGTTTTGCTGATCTCTGCAAC

>gac-mir-430a-44

TGGTCTTCATGGTTACCCTTACAAAAACACTGACTTTGGTTCTCAATACAGTAAGTGCTTTTTGTTGGGGTAGTTTT

>gac-mir-430a-45

TCAATGGAATCCAATTCAGATAACCTCAAACTGAGACACTGATGATTCTTCAGTTCATAAGTGCTTCTCTTTGGGGTTGTCTT

>gac-mir-430a-46

AACACTCTCAGATAACTTCAAATAGAGCCACTGGTGATGATTTGGTTCATAAGTGCTTCTCTTTGGGGTTGTCTTA

>gac-mir-430a-47

TTCTGTGTGTCCAGATTTGGCCATTAGGATTACCCTTACAAAAACACTGACTTTGGTTCTCAATACAGTAAGTGCTTTTTGTTGGGGTAGTTTTGCTGATCTCTGCAAC

>gac-mir-430a-48

TGGTCTTCATGGTTACCCTTACAAAAACACTGACTTTGGTTCTTAATACAGTAAGTGCTTTTTGTTGGGGTAGTTTTGCTGATCTCTGCAACA

>gac-mir-430a-49

TGGTCTTCATGGTTACCCTTACAAAAACACTGACTTTGGTTCTCAATACAGTAAGTGCTTTTTGTTGGGGTAGTTTT

>gac-mir-430a-5

TGGTCTTCATGGTTACCCTTACAAAAACACTGACTTTGATTCTCAATACAGTAAGTGCTTTTTGTTGGGGTAGTTTT

>gac-mir-430a-50

TCAATGGAATCCAATTCAGATAACCTCAAACTGAGACACTGATGATTCTTCAGTTCATAAGTGCTTCTCTTTGGGGTTGTCTT

>gac-mir-430a-51

AACACTCTCAGATAACTTCAAATAGAGCCACTGGTGATGATTTGGTTCATAAGTGCTTCTCTTTGGGGTTGTCTTA

>gac-mir-430a-52

TGTAGTGCGTGTCCAGATTTGGCCACTAGGATTACCCTTACAAAAACACTGACTTTAGTTCTCAACTCAGTAAGTGCTTTCTGTTGGGGTAGTTTTTCTGATCTCTGCAACTTCCACTATGTTAT

>gac-mir-430a-53

CAATATTCTCCATTGTGTCCTGATTTGGTCTTCATGGTTACCCTTACAAAAACACTGACTTTGGTTCTCAATACAGTAAGTGCTTTTTGTTGGGGTAGTTTTGCTGATCTCTGCAACA

>gac-mir-430a-54

TGGTCTTCATGGTTACCCTTACAAAAACACTGACTTTGGTTCTCAATACAGTAAGTGCTTTTTGTTGGGGTAGTTTTGCTGATCTCTGCAACA

>gac-mir-430a-55

TGGTCTTCATGGTTACCCTTACAAAAACACTGACTTTGGTTCTCAATACAGTAAGTGCTTTTTGTTGGGGTAGTTTT

>gac-mir-430a-6

TTCTGTGTGTCCAGATTTGGCCATTAGGATTACCCTTACAAAAACACTGACTTTGGTTCTCAATACAGTAAGTGCTTTTTGTTGGGGTAGTTTTGCTGATCTCTGCAAC

>gac-mir-430a-7

CAATATTCTCCATTGTGTCCTGATTTGGTCTTCATGGTTACCCTTACAAAAACACTGACTTTGGTTCTCAATACAGTAAGTGCTTTTTGTTGGGGTAGTTTTGCTGATCTCTGCAACA

>gac-mir-430a-8

TGGGCTTCATAGTTACCCTTACAAAAACACTGACTTTGGTTCTCAATACAGTAAGTGCTTTTTGTTGGGGTAGTTTT

>gac-mir-430a-9

TCAATGGAATCCAATTCAGATAACCTCAAACTGAGACACTGATGATTCTTCAGTTCATAAGTGCTTCTCTTTGGGGTTGTCTT

>gac-mir-430b-1

TCAATGGAATGCAATTCAGATAACCTCAAACTGAGACACTGATGATTCTTCAGTTCATAAGTGCTTCTCTTTGGGGTTGTCTT

>gac-mir-430b-10

TGGTCTTCATGGTTACCCTTACAAAAACACTGACTTTGGCTCTCAATACAGTAAGTGCTTTTTGTTGGGGTAGTTTT

>gac-mir-430b-11

TCAATGGAATCCAATTCAGATAACCTCAAACTGAGACACTGATGATTCTTCAGTTCATAAGTGCTTCTCTTTGGGGTTGTCTT

>gac-mir-430b-12

TTCTGTGTGTCCAGATTTGGCCATTAGGATTACCCTTACAAAAACACTGACTTTGGTTCTCAATACAGTAAGTGCTTTTTGTTGGGGTAGTTTTGCTGATCTCTGCAAC

>gac-mir-430b-13

CAATATTCTCCATTGTGTCCTGATTTGGTCTTCATGATTACCCTTACAAAAACACTGACTTTGGTTCTCAATACAGTAAGTGCTTTTTGTTGGGGTAGTTTTGCTGATCTCTGCAACA

>gac-mir-430b-14

TGGTCTTCATGGTTACCCTTACAAAAACACTGACTTTGGTTCTCAATACAGTAAGTGCTTTTTGTTGGGGTAGTTTT

>gac-mir-430b-15

TCAATGGAATCCAATTCAGATAACCTCAAACTGAGACACTGATGATTCTTCAGTTCATAAGTGCTTCTCTTTGGGGTTGTCTT

>gac-mir-430b-16

TTCTGTGTGTCCAGATTTGGCCATTAGGATTACCCTTACAAAAACACTGACTTTGGTTCTCAATACAGTAAGTGCTTTTTGTTGGGGTAGTTTTGCTGATCTCTGCAAC

>gac-mir-430b-17

TGGTCTTCATGGTTACCCTTACAAAAACACTGACTTTGGTTCTCAATACAGTAAGTGCTTTTTGTTGGGGTAGTTTT

>gac-mir-430b-18

TTCTGTGTGTCCAGATTTGGCCATTAGGATTACCCTTACAAAAACACTGACTTTGGTTCTCAATACAGTAAGTGCTTTTTGTTGGGGTAGTTTTGCTGATCTCTGCAAC

>gac-mir-430b-19

CAATATTCTCCATTGTGTCCTGATTTGGTCTTCATGGTTACCCTTACAAAAACACTGACTTTGGTTCTCAATACAGTAAGTGCTTTTTGTTGGGGTAGTTTTGCTGATCTCTGCAACA

>gac-mir-430b-2

TTCTGTGTGTCCAGATTTGGCCATTAGGATTACCCTTACAAAAACACTGACTTTGGTTCTCAATACAGTAAGTGCTTTTTGTTGGGGTAGTTTTGCTGATCTCTGCAAC

>gac-mir-430b-20

TGGTCTTCATGGTTACCCTTACAAAAACACTGACTTTGGTTCTTAATACAGTAAGTGCTTTTTGTTGGGGTAGTTTTGCTGATCTCTGCAACA

>gac-mir-430b-21

TGGTCTTCATGGTTACCCTTACAAAAACACTGACTTTGGTTCTCAATACAGTAAGTGCTTTTTGTTGGGGTAGTTTT

>gac-mir-430b-22

TCAATGGAATCCAATTCAGATAACCTCAAACTGAGACACTGATGATTCTTCAGTTCATAAGTGCTTCTCTTTGGGGTTGTCTT

>gac-mir-430b-23

TTTCTCCTCCAGATTTGGTCATTAGGATTACCCTTACAAAAACACTGACTTTGGTTCTCAATACAGTAAGTGCTTTTTGTTGGGGTAGTTTTGCTGATCTCTGCAACA

>gac-mir-430b-24

TCAATGGAATCCAATTCAGATAACCTCAAACTGAGACACTGATGATTCTTCAGTTCATAAGTGCTTCTCTTTGGGGTTGTCTT

>gac-mir-430b-25

TGGTCTTCATGGTTACCCTTACAAAAACACTGACTTTGGTTCTTAATACAGTAAGTGCTTTTTGTTGGGGTAGTTT

>gac-mir-430b-26

GTCTTCCAGATTTTGGCCATTAGGATTACCCTTACAAAAACACTGACTTTGGTTCTCAATACAGTAAGTGCTTTTTGTTGGGGTAGTTTTGCTGATCTCTGCAAC

>gac-mir-430b-27

CAATATTCTCCATTGTGTCCTGATTTGGTCTTCATGGTTACCCTTACAAAAACACTGACTTTGGTTCTCAATACAGTAAGTGCTTTTTGTTGGGGTAGTTTTGCTGATCTCTGCAACA

>gac-mir-430b-28

TGGTCTTCATGGTTACCCTTACAAAAACACTGACTTTGGTTCTTAATACAGTAAGTGCTTTTTGTTGGGGTAGTTTTGCTGATCTCTGCAACA

>gac-mir-430b-29

TGGTCTTCATGGTTACCCTTACAAAAACACTGACTTTGGTTCTCAATACAGTAAGTGCTTTTTGTTGGGGTAGTTTT

>gac-mir-430b-3

CAATATTCTCCATTGTGTCCTGATTTGGTCTTCATGGTTACCCTTACAAAAACACTGACTTTGGTTCTCAATACAGTAAGTGCTTTTTGTTGGGGTAGTTTTGCTGATCTCTGCAACA

>gac-mir-430b-30

TGGTCTTCATGGTTACCCTTACAAAAACACTGACTTTGGTTCTCAATACAGTAAGTGCTTTTTGTTGGGGTAGTTTT

>gac-mir-430b-31

TTAATGGAATCCAATTCAGATAACCTCAAAATGAGACACTGATGATTCTTCAGTTCATAAGTGCTTCTCTTTGGGGTTGTCTT

>gac-mir-430b-32

TGGTCTTCATGGTTACCCTTACAAAAACACTGACTTTGGTTCTCAATACAGTAAGTGCTTTTTGTTGGGGTAGTTTT

>gac-mir-430b-33

TCAATGGAATCCAATTCAGATAACCTCAAACTGAGACACTGATGATTCTTCAGTTCATAAGTGCTTCTCTTTGGGGTTGTCTT

>gac-mir-430b-34

TTTCTCCTCCAGATTTGGCCATTAGGATTACCCTTACAAAAACACTGACTTTGGTTCTCAATACAGTAAGTGCTTTTTGTTGGGGTAGTTTTGCTGATCTCTGCAACA

>gac-mir-430b-35

TGGTCTTCATGGTTACCCTTACAAAAACACTGACTTTGGTTCTCAATACAGTAAGTGCTTTTTGTTGGGGTAGTTTT

>gac-mir-430b-36

TCAATGGAATCCAATTCAGATAACCTCAAACTGAGACACTGATGATTCTTCAGTTCATAAGTGCTTCTCTTTGGGGTTGTCTT

>gac-mir-430b-37

TGGTCTTCATGGTTACCCTTACAAAAACACTGACTTTGGTTCTCAATACAGTAAGTGCTTTTTGTTGGGGTAGTTTT

>gac-mir-430b-38

TCAATGGAATCCAATTCAGATAACCTCAAACTGAGACACTGATGATTCTTCAGTTCATAAGTGCTTCTCTTTGGGGTTGTCTT

>gac-mir-430b-39

TGGTCTTCATGGTTACCCTTACAAAAACACTGACTTTGGTTCTCAATACAGTAAGTGCTTTTTGTTGGGGTAGTTTT

>gac-mir-430b-4

TGGTCTTCATGGTTACCCTTACAAAAACACTGACTTTGGTTCTCAATACAGTAAGTGCTTTTTGTTGGGGTAGTTTT

>gac-mir-430b-40

TTAATGGAATGCAATTCAGATAACCTCAAAATGAGACACTGATGATTCTTCAGTTCATAAGTGCTTCTCTTTGGGGTTGTCTT

>gac-mir-430b-41

TCAATGGAATCCAATTCAGATAACCTCAAACTGAGACACTGATGATTCTTCAGTTCATAAGTGCTTCTCTTTGGGGTTGTCTT

>gac-mir-430b-42

TTCTGTGTGTCCAGATTTGGCCATTAGGATTACCCTTACAAAAACACTGACTTTGGTTCTCAATACAGTAAGTGCTTTTTGTTGGGGTAGTTTTGCTGACCTCTGCAAC

>gac-mir-430b-43

TACCCTTACAAAAACACTGACTTTGGTTCTCAATACAGTAAGTGCTTTTTGTTGGGGTAGTTTTGCTGATCTCTGCAACA

>gac-mir-430b-44

TGGTCTTCATGGTTACCCTTACAAAAACACTGACTTTGGTTCTCAATACAGTAAGTGCTTTTTGTTGGGGTAGTTTT

>gac-mir-430b-45

TTCTGTGTGTCCAGATTTGGCCATTAGGATTACCCTTACAAAAACACTGACTTTGGTTCTCAATACAGTAAGTGCTTTTTGTTGGGGTAGTTTTGCTGATCTCTGCAACA

>gac-mir-430b-46

TGGTCTTCATGGTTACCCTTACAAAAACACTGACTTTGGTTCTCAATACAGTAAGTGCTTTTTGTTGGGGTAGTTTTGCTGATCTCTGCAACA

>gac-mir-430b-47

TGGTCTTCATGGTTACCCTTACAAAAACACTGACTTTGGTTCTCAATACAGTAAGTGCTTTTTGTTGGGGTAGTTTT

>gac-mir-430b-48

TTCTGTGTGTCCAGATTTGGCCATTAGGATTACCCTTACAAAAACACTGACTTTGGTTCTCAATACAGTAAGTGCTTTTTGTTGGGGTAGTTTTGCTGATCTCTGCAAC

>gac-mir-430b-49

TGGTCTTCATGGTTACCCTTACAAAAACACTGACTTTGGTTCTTAATACAGTAAGTGCTTTTTGTTGGGGTAGTTTTGCTGATCTCTGCAACA

>gac-mir-430b-5

TCAATGGAATCCAATTCAGATAACCTCAAACTGAGACACTGATGATTCTTCAGTTCATAAGTGCTTCTCTTTGGGGTTGTCTT

>gac-mir-430b-50

TGGTCTTCATGGTTACCCTTACAAAAACACTGACTTTGGTTCTCAATACAGTAAGTGCTTTTTGTTGGGGTAGTTTT

>gac-mir-430b-51

TCAATGGAATCCAATTCAGATAACCTCAAACTGAGACACTGATGATTCTTCAGTTCATAAGTGCTTCTCTTTGGGGTTGTCTT

>gac-mir-430b-52

TGGTCTTCATGGTTACCCTTACAAAAACACTGACTTTGGTTCTCAATACAGTAAGTGCTTTTTGTTGGGGTAGTTTT

>gac-mir-430b-53

AACACTCTCAGATAACTTCAAACAGAGCCACTGGTGATGATTTAGTTCATAAGTGCTTCTCTTTGGGGTTGTCTTA

>gac-mir-430b-54

TGGTCTTCATGGTTACCCTTACAAAAACACTGACTTTGGTTCTCAATACAGTAAGTGCTTTTTGTTGGGGTAGTTTT

>gac-mir-430b-55

TCAATGGAATCCAATTCAGATAACCTCAAACTGAGACACTGATGATTCTTCAGTTCATAAGTGCTTCTCTTTGGGGTTGTCTT

>gac-mir-430b-56

CAATATTCTCCATTGTGTCCTGATTTGGTCTTCATGGTTACCCTTACAAAAACACTGACTTTGGTTCTCAATACAGTAAGTGCTTTTTGTTGGGGTAGTTTTGCTGATCTCTGCAACA

>gac-mir-430b-57

TGGTCTTCATGGTTACCCTTACAAAAACACTGACTTTGGTTCTCAATACAGTAAGTGCTTTTTGTTGGGGTAGTTTT

>gac-mir-430b-58

TCAATGGAATGCAATTCAGATAACCTCAAAATGAGACACTGATGATTCTTCAGTTCATAAGTGCTTCTCTTTGGGGTTGTCTT

>gac-mir-430b-59

TGGTCTTCATGGTTACCCTTACAAAAACACTGACTTTGGTTCTCAATACAGTAAGTGCTTTTTGTTGGGGTAGTTTT

>gac-mir-430b-6

TTCTGTGTGTCCAGATTTGGCCATTAGGATTACCCTTACAAAAACACTGACTTTGGTTCTCAATACAGTAAGTGCTTTTTGTTGGGGTAGTTTTGCTGATCTCTGCAAC

>gac-mir-430b-60

TCAATGGAATCCAATTCAGATAACCTCAAACTGAGATACTGATGATTCTTCAGTTCATAAGTGCTTCTCTTTGGGGTTGTCTT

>gac-mir-430b-61

TTTCTCCTCCAGATTTGGCCATTAGGATTACCCTTACAAAAACACTGACTTTGGTTCTCAATACAGTAAGTGCTTTTTGTTGGGGTAGTTTTGCTGATCTCTGCAACA

>gac-mir-430b-62

TGGTCTTCATGGTTACCCTTACAAAAACACTGACTTTGGTTCTCAATACAGTAAGTGCTTTTTGTTGGGGTAGTTTT

>gac-mir-430b-63

TCAATGGAATCCAATTCAGATAACCTCAAAATGAGACACTGATGATTCTTCAGTTCATAAGTGCTTCTCTTTGGGGTTGTCTT

>gac-mir-430b-64

TTCTGTGTGTCCAGATTTGGCCATTAGGATTACCCTTACAAAAACACTGACTTTGGTTCTCAATACAGTAAGTGCTTTTTGTTGGGGTAGTTTTGCTGATCTCTGCAAC

>gac-mir-430b-65

TGGTCTTCATGGTTACCCTTACAAAAACACTGACTTTGGTTCTTAATACAGTAAGTGCTTTTTGTTGGGGTAGTTT

>gac-mir-430b-66

TCAATGGAATCCAATTCAGATAACCTCAAACTGAGACACTGATGATTCTTCAGTTCATAAGTGCTTCTCTTTGGGGTTGTCTT

>gac-mir-430b-67

TGGTCTTCATGGTTACCCTTACAAAAACACTGACTTTGGTTCTCAATACAGTAAGTGCTTTTTGTTGGGGTAGTTTT

>gac-mir-430b-68

AATGGAATCCAATTCAGATCACCTCAAAATGAGACACTGTTGATTCTTCAGATCATAAGTGCTTCTCTTTGGGGTTGTCTT

>gac-mir-430b-69

TTCTGTGTGTCCAGATTTGGCCATTAGGATTACCCTTACAAAAACACTGACTTTGGTTCTCAATACAGTAAGTGCTTTTTGTTGGGGTAGTTTTGCTGATCTCTGCAAC

>gac-mir-430b-7

TGGTCTTCATGGTTACCCTTACAAAAACACTGACTTTGGTTCTCAATACAGTAAGTGCTTTTTGTTGGGGTAGTTTT

>gac-mir-430b-70

TGGTCTTCATGGTTACCCTTACAAAAACACTGACTTTGGTTCTCAATACAGTAAGTGCTTTTTGTTGGGGTAGTTTT

>gac-mir-430b-71

TCAATGGAATGCAATTCAGATAACCTCAAAATGAGACACTGATGATTCTTCAGTTCATAAGTGCTTCTCTTTGGGGTTGTCTT

>gac-mir-430b-72

TGGTCTTCATGGTTACCCTTACAAAAACACTGACTTTGGTTCTCAATACAGTAAGTGCTTTTTGTTGGGGTAGTTTT

>gac-mir-430b-73

TCAATGGAATCCAATTCAGATAACCTCAAACTGAGATACTGATGATTCTTCAGTTCATAAGTGCTTCTCTTTGGGGTTGTCTT

>gac-mir-430b-74

TCAATGGAATCCAATTCAGATAACCTCAAACTGAGACACTGATGATTCTTCAGTTCATAAGTGCTTCTCTTTGGGGTTGTCTT

>gac-mir-430b-75

TACCCTTACAAAAACACTGACTTTGGTTCTCAATACAGTAAGTGCTTTTTGTTGGGGTAGTTTTGCTGATCTCTGCAACA

>gac-mir-430b-76

TGGTCTTCATGGTTACCCTTACAAAAACACTGACTTTGGTTCTCAATACAGTAAGTGCTTTTTGTTGGGGTAGTTTTGCTGATCTCTGCAACA

>gac-mir-430b-77

TGGTCTTCATGGTTACCCTTACAAAAACACTGACTTTGGTTCTCAATACAGTAAGTGCTTTTTGTTGGGGTAGTTTT

>gac-mir-430b-78

TCAATGGAATCCAATTCAGATAACCTCAAACTGAGACACTGATGATTCTTCAGTTCATAAGTGCTTCTCTTTGGGGTTGTCTT

>gac-mir-430b-79

CAATATTCTCCATTGTGTCCTGATTTGGTCTTCATGGTTACCCTTACAAAAACACTGACTTTGTTTCTCAAATACAGTAAGTGCTTTTTGTTGGGGTAGTTTTGCTGATCTCTGCAACA

>gac-mir-430b-8

TCAATGGAATCCAATTCAGATAACCTCAAACTGAGACACTGATGATTCTTCAGTTCATAAGTGCTTCTCTTTGGGGTTGTCTT

>gac-mir-430b-80

TGGTCTTCATGGTTACCCTTACAAAAACACTGACTTTGGTTCTCAATACAGTAAGTGCTTTTTGTTGGGGTAGTTTTGCTGATCTCTGCAACA

>gac-mir-430b-81

TGGTCTTCATGGTTACCCTTACAAAAACACTGACTTTGGTTCTCAATACAGTAAGTGCTTTTTGTTGGGGTAGTTTT

>gac-mir-430b-82

CAATATTCTCCATTGTGTCCTGATTTGGTCTTCATGGTTACCCTTACAAAAACACTGACTTTGGTTCTCAATACAGTAAGTGCTTTTTGTTGGGGTAGTTTTGCTGATCTCTGCAACA

>gac-mir-430b-83

TCAATGGAATCCAATTCAGATAACCTCAAAATGAGACACTGATGATTCTTCAGTTCATAAGTGCTTCTCTTTGGGGTTGTCTT

>gac-mir-430b-84

TTTCTCCTCCAGATTTGGCCATTAGGATTACCCTTACAAAAACACTGACTTTGGTTCTCAATACAGTAAGTGCTTTTTGTTGGGGTAGTTTTGCTGATCTCTGCAACA

>gac-mir-430b-9

TTTCTCTTCCAGATTTGGCCATTAGGATTACCCTTACAAAAACACTGACTTTGGTTCTCAATACAGTAAGCGCTTTTTGTTGGGGTAGTTTTGCTGATCTCTGCAACA

>gac-mir-449

TGTGGGCGGCTGGTAGGCAGTGTCTTGTTAGCTGGTCAATTCTGCGCCCCAGCTTACCTGCTGCTGCCCTCCAGCTGTCCAT

>gac-mir-451

AGAGGCCGTCAAACCGTTACCATTACTGAGTTTCAGTTATGGTAAGGGTTATACGGCCCT

>gac-mir-454b

TGCCCTCGCAGATGAGACCCTATCAATGTTGCCTCTGCTTTCACTTGAGTCAACAGAGTAGTGCAATATTGCTTATAGGGTCTTGCCTGTGAGG

>gac-mir-455a

TCCCTGAGGTGAGGGTATGTGCCCTTGGACTACATCGTGGAAGCCAGCACCATGCAGTCCATGGGCATATACACTTGCCTCAAGGCTTGGCT

>gac-mir-455b

TCCCTGGCGTGAGGGTATGTGCCCTTGGACTACATCGTGGAAGCCAGCACCATGCAGTCCATGGGCATATACACTTGCCTCATGGCTTGTTTCTTC

>gac-mir-456

GCTGGCTCTGTGAGCAGGCATCTTCCTAGCCTACATGTAGATCCAGGAATCTGCAGGCTGGTTAGATGGTTGTCACATGTCTAGC

>gac-mir-458

CTCCGCCTCCTGCAATCGGTGTGGACGGCAGCGCCATTTTCAGAGCTATAAGTGTGAAGTGGTCATAGCTCTTTAAATGGTACTGCCGTGTGCACTCAACAGACCAGC

>gac-mir-459

GCCGTCAGTAACAAGGATTCATCCTGTTGTGATGCTGCAGGCTGACAGGATATCGTTGTTACTGGGGGCGAGGCT

>gac-mir-460

CAGCTCCTGCATTGTACACACTGTGCGTAGAAGTTACGTAAGCACAGCGCATACAATGTGGATGCTGTG

>gac-mir-489

GTGGTGGCCTGGTGGTCGTATGTATGACGTCATTTACTTCAATGTTTGGAGTGACATCATATGTACGGCTGCTAAACTGCTACATGGGACATCA

>gac-mir-499a

CTGAGACGGAGGCAGTTAAGACTTGCAGTGATGTTTAGGGCAATGATCACATGAACATCACTCTAAGTCTGTGCTGGCTCCTCT

>gac-mir-551a

CCTTAGAAACTAAGCGTGGGTGTGGCCTGGAAAAGAAACACGGCGACCCATCCTTAGTTTCTGAGG

>gac-mir-705

GCATCAGGTGGGAAGTGGGGTGGTTGCCGAAGTCCGAGCTGACGTAGCCAATACGCAGTCGACCC

>gac-mir-7132a

TCTGCTCCTAGACTTGGTCAAAGCTCCTCAGTGTTGTTTAGGAACCTGGGGAGCTTAGAACAAGTGTAAGA

>gac-mir-7132b

TTTGCCCCTGGACTTGGTCAAAGCTCCTCAGCAGATTAAAAGAGACTCTGAGGCGTTTGGAACAAGTTCAG

>gac-mir-7133

TTTGGAAATGGCTCAGATGTTGAGTGTCAAACTGTATGTTGTATATGTCGTATATAGTTTGATACACAGCACAATAAGCC

>gac-mir-7147

GAGTGCCCAGTGCTGTACCATGCTGGTAGCCAGTGTGTGGTAGGCTCTGCTGGTTGACCAGTGTTGTGCCTCACTGGCTGCTC

>gac-mir-722a

GGAACAGAGTGGAATTTGAAACGTTTTCGCCAAAAATGTTTCCATCGTCAAGGTGTTTTTTGCAGAAACGTTTCAGATTTCGTTTTGTTCT

>gac-mir-722b

GAACAGAATGGAATTTGAAACGTTTTAGCCAAAAATGTTTCTATGGTCAAGGTGTTTTTTGCAGAAACGTTTCAGATTTCCTTCCGTTCT

>gac-mir-723a

CGGCAGCTTTGTATGATGTTACTTCTGACGTATCGGAGAAGACATCAGATAAATCTGTGCT

>gac-mir-723b

AGGCAGTTTTGATGATGTTACTTTTTCTATTTTAAGAAGACATCAGATAAATCTGTGCT

>gac-mir-724a

CAGCAGACTGGATTTAAAGGGAATTTGCGACTGTTAGTCAAAACATTAGAACAGCCACACCTTCCTTTTAAGATCTTGCCTGCTG

>gac-mir-724b

CAGCAGACTGGGTTTAAAGGGAATTTGCGACTGTTAATCAAACCAATCGAACAGCCACACCTTCCTTTTAAGATCTTGTCTGCTG

>gac-mir-725

CACTGCTGGAAACCTTGCCTGTGAATTTCTATTGAAAATTTCAGTCATTGTTTCTGGTCGT

>gac-mir-726

GTAGGTCTGGAATTCCGCTAGTTCTGAACTATTCGGGATTGGTAAAAGTTCACTACTAGCAGAACTCAGATTTGCTCCCGA

>gac-mir-727b

CTGTATGTCATTTTCAGTCTTCAATTCCTCCCAGCCCGTTACTATGGAAACTGTGAGTTGAGGCGAGTTGAAGACTAAAAATGCTGTACAG

>gac-mir-728a

GAGAAATGTAGTAGACTTAAAGTATACGTGTGGAGCCAGAGAGTATACTAAGTACACTACGTTTAT

>gac-mir-728b

CTTATGAGAAAATGTAGTAGACTATAAGTATACCTGTACCTGTATGTATACTAAGTACACTACGTTTACTCAAGAG

>gac-mir-729

CCATCATCAAAGCTGGGGCTGTATCATAACCAGTCTGGTTATTTTAGAAGCATGGGTATGATACGACCTCAGTT

>gac-mir-730

GCGTGCTCGGGGCCTCCTCATTGTGCATGCTGTGTGTGTTTCTGTGAGTGCCACACAGCGCCTGCAATGTGGAGGCT

>gac-mir-733-1(vdmiR)

GCATCGGTTTAGCTCAGTGGTTACTTCCAAACATGAGTTTCTTTCTTCACGGGCTCTTCTGGGGCCCATCGATCGACCACGGGCGCTGTCCGATGCTTTT

>gac-mir-733-2(vdmiR)

GCGTCGGTTTAGCTCAGTGGTTACTTCTACAATCCACGTATCTTTCTCTGACGGGCTGCTGTCCGTCGGAGATCGACCACGGGCGCTGTCCGATGCTTTT

>gac-mir-733-3(vdmiR)

GCGTCGGTTTAGCTCAGTGGTTACTTCTGGTCATGTGTTTGCCTTCCCTCGGGCTCTTCTGGGGCCCTGAGTTCGACCACGGGCGCTGTCCGATGCATTT

>gac-mir-734

TTTCAAGAGCTGAACTATTCTGCAACATTTGTTGATGTGGATCTGTAAGTAAATGCTGCAGAATTGTGCTCCTCTTGGAAT

>gac-mir-736-1

CTAGCAGATCAGCTTTTTGTTTGTGTTATGTTCATACTAAAATGTAAGACGAACAAAAAGTTTGTCTGTTGG

>gac-mir-737

ATCTGCCGCTCTGTGGTTTTTTTAGGTTTTGATTTTTGGACTTGGATGAGAAAATCTAAATCTAAAGAAAATACAGCGCTGACGAGA

>gac-mir-7552a

CTTACAATTAAAGGATATTTCTCGTGACTGCAAAATAAAAAACGGAAATATCTCTTAATTGTTTGGTTAAGTCTGTCAG

>gac-mir-7a-1

GACGCGCTGTGGAAGACTAGTGATTTTGTTGTTCTAAGCTCACGTCCCTGACAACAAGTCCCAGTCTACCTCAGCGAGTCCCGCTA

>gac-mir-7a-3

TGCTGAGTGGAAGACTAGTGATTTTGTTGTGAAGCTGATCAAAGAACAACAAGTCCCAGTCTACCTCTCAGCAC

>gac-mir-7a-4

TGGAAGACTAGTGATTTTGTTGTTTTGTTGTTAAACCGACAACAAGTCATTGTCTCCCGCGCTGC

>gac-mir-7bb

CCCTGGCTGCTGCTGTGTGGAAGACTAGTGATTTTGTTGTTTTTAGTTAGATCAACCGACAACAAATCACAGTCTGCCAAATAGCACAGGC

>gac-mir-8159

TCCCCCAGGCTCAGTAACTGGAATCTGTCCCTGCAGAGTGTAAAATCGCAGGGCCCGTCTGGTTACTGCACCTGGGGGCCAAGCTTCT

>gac-mir-8160a

TTGCTGGGGCGGTCAGAATAATGCCAGCAGTCGGTCACGGTCTCCCAGGGACCAGCACTGGTGTTATTGGGACTGACTCAGTC

>gac-mir-8160b

CGTGCTGGTCAGAATAATGCCAGCAGTCGGCCATGCTGTCCGTCTCAGGCCAGCGCTGGTGTTATTGAGTCCAACACGGA

>gac-mir-9-1a

CTGAGGGGTTGTCTGTTATCTTTGGTTATCTAGCTGTATGAGTGGCGTACATTCTTCATAAAGCTAGATAACCGAAAGTAACAAGAATCCCATTA

>gac-mir-9-1b

AGGGGTTGGTTGTTATCTTTGGTTATCTAGCTGTATGAGTGATGTACATTCTTCATAAAGCTAGATAACCGAAAGTAACAAGAATCCCATTA

>gac-mir-9-3

GGGGGTCTGTTTCTGTCTTTGGTTATCTAGCTGTATGAGTTTTAAATGCCTGTCATAAAGCTAGATAACCGAAAGTAGAAAT

>gac-mir-9-5

GTTGTTATCTTTGGTTATCTAGCTGTATGAGTGTTCTGCTCGTCATAAAGCTAGATAACCGAAAGTAAAAAC

>gac-mir-9-6

GGAGCTTGTTCCTGTCTTTGGTTATCTAGCTGTATGAGTGTTAATCATCCATCATAAAGCTAGATAACCGAAAGTAGGAATGGCCTCC

>gac-mir-9-7

GGGTTAGTTCGTGTCTTTGGTTATCTAGCTGTATGAGTTTTAATTTCATAAAGCTAGAGAACCGAAAGTACGAACTGACGCC

>gac-mir-92a-1

GTCCCTTCCTGTGCAGGTGGGGATTAGTAGCAATGCTGTGTACCAGAAGGTATTGCACTTGTCCCGGCCTGTATAGGA

>gac-mir-92a-2

CTTTCTGAGCAGGTTGGGAGAGGTAGCAATGCTCTGTACACATGTGGTATTGCACTTGTCCCGGCCTGTTGAGGA

>gac-mir-96a

CTCTTCTTTGCCCATTTTGGCACTAGCACATTTTTGCTTCTGTATATATACTTTGAGCAATTATGTGTAGTGCCAATATAGGACAAGACAGACTTCCATC

>gac-mir-96b

TCTCCTTGGCCCGTTTTGGCACTAGCACATTTTTGCTTTGTCCCTTGTTTGTTTGAGCAAACATGTGTGGTGCCAATTTAGGACAAGGCAGA

>gac-mir-99a

CCATTTGGCACAAACCCGTAGATCCGATCTTGTGGCAAATCTGACAGCACAAGCTCGCCTCTGTGGGTCTTTGTCATTGTGGT

>gac-mir-ENSGACG00000022167

ATTGCCCGGATGAGCCTCGGTGGTCCGGGGTGCAGGCTTCAAACCTGTAGCTGCTTAGCGGCAGAGTGGTTCAATTCCACCTTTCGGGCG

>gac-mir-Rastorguev366

CGTTACTTTACCCGGCGAAGTAACGTTAGCTTGCGAAGCTAGCATACGAAGCTAACGTTAGTGTCGCTGGACAAGGTGACGT

>gac-mir-Rastorguev443

ACACCGTACCGGTCAAAGGTTTGGACACACGAGTCAATGAGAAAGTGTGTCCCAATTTCAGACCGGCACTGTGCGT

>gac-mir-Rastorguev458

GCTTTGCGGGCGCTGCGTTCGGTGCCTCGTCCGGTTCGGTGCGGCTGAGGTACCAGAACGCCGGCCCGGCGGGCTGG

Supplementary File 6: Stickleback mature miRNA annotation.

>gac-let-7a-1-3p

CTATTAAACCTACTGTCTTTCC

>gac-let-7a-3/7-3p

CTATACAACTTACTGTCTTTCC

>gac-let-7a-4-3p

CTGTACAGCCTCCTAGCTTTCC

>gac-let-7a-5-3p

CTATACAGTCTATTGCCTTCCC

>gac-let-7a-5p

TGAGGTAGTAGGTTGTATAGTT

>gac-let-7a-6-3p

CTACACAGCCTATTACCTTCCT

>gac-let-7b-1-3p

CTATACAACCTACTGCCTTTC

>gac-let-7b-2-3p

CTATACAACATACTGCCTTCCC

>gac-let-7b-5p

TGAGGTAGTAGGTTGTGTGGTT

>gac-let-7c-2-5p

TGAGGTAGTAGGTTGTATGGTT

>gac-let-7c-2/d-3p

CTGTACAACCTTCTAGCTTTCC

>gac-let-7d-5p

TGAGGTAGTTGGTTGTATGGTT

>gac-let-7e-1-3p

CTATACAATCTACTGTCTTTCC

>gac-let-7e-2/g-2-3p

CTATACAGTCTACTGTCTTTCC

>gac-let-7e-5p

TGAGGTAGTAGATTGAATAGTT

>gac-let-7f-3p

CTATACAATCTATTGCCTTCC

>gac-let-7f-5p

TGAGGTAGTAGATTGTATAGTT

>gac-let-7g-1-3p

CTATACAGCCTACTGTCTTTCT

>gac-let-7g-5p

TGAGGTAGTAGTTTGTATAGTT

>gac-let-7h-1-3p

CTATACAACTTACTGCCTTCCT

>gac-let-7h-1-5p

TGAGGTAGTAAGTTGTGTTGTT

>gac-let-7h-2-3p

CTATACAACATACTGCCTTAC

>gac-let-7h-2-5p

TGAGGTAGTATGTTGTGTTGTT

>gac-let-7i-1-3p

CTGCGCAAGCTACTGCCTTGC

>gac-let-7i-5p

TGAGGTAGTAGTTTGTGCTGTT

>gac-let-7j-3p

CTGTACAAGTGACTGCCTTGC

>gac-let-7j-5p

TGAGGTAGTTGTTTGTACAGTT

>gac-miR-1-2-5p

ACATACTTCTTTATGTACCCAT

>gac-miR-1-3p

TGGAATGTAAAGAAGTATGTAT

>gac-miR-100-2-3p

CAAGCTCGTGTCTATAGGTATG

>gac-miR-100-2-5p

AACCCGTAGATCCGAACTTGT

>gac-miR-101a-3p

TACAGTACTGTGATAACTGAAG

>gac-miR-101a-5p

TCAGTTATCACAGTGCTGATGC

>gac-miR-101b-3p

TACAGTACTATGATAACTGAAG

>gac-miR-101b-5p

TCAGTTATCATGGTACCGGTGC

>gac-miR-103-3p

AGCAGCATTGTACAGGGCTATGA

>gac-miR-103a-5p

AGCCTCTTTACGGTGCTGCCTTG

>gac-miR-103b-5p

AGCCTCTTTACAGTGCTGCCTTG

>gac-miR-10544-3p

TGCGCACGGGGCCACGCCCTGC

>gac-miR-10544-5p

TAGGCGTGTCACTGCGTGTCAC

>gac-miR-10545-3p

CTGCTCAGGTGTGGGACCATGA

>gac-miR-10545-5p

TAAGTCTCACACCAGTGCAAAAC

>gac-miR-10b-1-3p

CAAATTCGATTCTAGGGGAGT

>gac-miR-10b-2-3p

CAAATAGGTCTCTGCAGGAATA

>gac-miR-10b-5p

TACCCTGTAGAACCGAATTTGT

>gac-miR-10c-3p

CAAATTCGCTTCTAGGGGAGT

>gac-miR-10c-5p

TACCCTGTAGATCCGGATTTGT

>gac-miR-122-3p

AACGCCATTATCACACTAAAT

>gac-miR-122-5p

TGGAGTGTGACAATGGTGTTT

>gac-miR-124-3-5p

CGTGTTCACAGCGGACCTTGAT

>gac-miR-124-3p

TAAGGCACGCGGTGAATGCC

>gac-miR-124-4a-5p

TGTGTTCACAGTGGACCTTGAT

>gac-miR-124-4b-5p

AGTGTTCACAGTGGACCTTGA

>gac-miR-124-6-5p

CGTGTTCACGGCGGACCTTGA

>gac-miR-125a-1-3p

ACAGGTGAGGTCCTTGGGAAC

>gac-miR-125a-2-3p

CAGGTGAGGTCCTTGGGAAT

>gac-miR-125a-5p

TCCCTGAGACCCTTAACCTGTG

>gac-miR-125b-2-3p

ACGGGTTGGGTTCTTGGGAGCT

>gac-miR-125b-2/3-5p

TCCCTGAGACCCTAACTTGTG

>gac-miR-125b-3-3p

ACGGGTTAGGCTCTCGGGACGC

>gac-miR-126b-3p

TCGTACCGTGAGTAATAATGCA

>gac-miR-126b-5p

CATTATTACTTTTGGTACGCG

>gac-miR-128-2-3p

TCACAGTGAACCGGTCTCTTT

>gac-miR-128-2-5p

GGGGGCCGTTACACTGTCAGA

>gac-miR-129-1a-3p

AAGCCCTTACCCCAAAAAGTAT

>gac-miR-129-1a/2-5p

CTTTTTGCGGTCTGGGCTTGC

>gac-miR-129-1b-5p

CTTTTTGCGGTCTGGACTTGC

>gac-miR-129-1b/2-3p

AAGCCCTTACCCCAAAAAGCAT

>gac-miR-1306-5p

CACCTCCCCTGCAAACGTCC

>gac-miR-130a-3p

CAGTGCAACGTTAAAAGGGCAT

>gac-miR-130b-3p

CAGTGCAATAATGAAAGGGCA

>gac-miR-130b-5p

ACTCTTTCCCTGTTGCACTACT

>gac-miR-130c-1-5p

GCCCTTTTCCTCTTGCACTACT

>gac-miR-130c-2-5p

GCCCTTTTTCTGTTGTACTAC

>gac-miR-130c-3p

CAGTGCAATATTAAAAGGGCA

>gac-miR-132-3p

TAACAGTCTACAGCCATGGTC

>gac-miR-132a-5p

ACCGTGGCATTAGATTGTTA

>gac-miR-132b-5p

ACCGTGGCTTTAGATTGTTACT

>gac-miR-133a-3p

TTGGTCCCCTTCAACCAGCTGT

>gac-miR-133a-5p

AGCTGGTAAAATGGAACCAAAT

>gac-miR-133b-3p

TTTGGTCCCCTTCAACCAGCTA

>gac-miR-135a-1-3p

TGTAGGAGTAGAAGCCACT

>gac-miR-135a-1-5p

TATGGCTTTTTATTCCTACGTGA

>gac-miR-135a-2-3p

GTGTAGGAACAGAAGCCATTTT

>gac-miR-135a-2-5p

TATGGCTTTTTATTCCTATGTGA

>gac-miR-135b-3p

TATAGGGATGGAAGCCATGCA

>gac-miR-135b-5p

TATGGCTTTTTATTCCTATCTG

>gac-miR-135c-5p

TATGGCTTTCTATTCCTATGTGA

>gac-miR-137-1-3p

TATTGCTTGAGAATACGCGTAG

>gac-miR-137-1-5p

CACGGGTATTCTTGGGTTGAT

>gac-miR-137-2a-3p

TATTGCTTAAGAATACGCGTAG

>gac-miR-137-2a-5p

CTGAAAGACTCTCTTCGGTG

>gac-miR-138-5p

AGCTGGTGTTGTGAATCAGGCCG

>gac-miR-1388-3p

ATCTCAGGTTCGTCAGCCCACG

>gac-miR-1388-5p

AGGACTGTCCGACCTGAGAATT

>gac-miR-138a-3p

GCTATTTCACAACACCAGGGT

>gac-miR-138ba-3p

GCTTCTTCACAACACCAGGGT

>gac-miR-138bb-3p

GCTACTTCCCAACACCAGGGT

>gac-miR-139-3p

TGGAGACTCAGCTCTGTTGGAAT

>gac-miR-139-5p

TCTACAGTGCATGTGTCTCCAGT

>gac-miR-140-3p

TACCACAGGGTAGAACCACGGA

>gac-miR-140-5p

CAGTGGTTTTACCCTATGGTAG

>gac-miR-142-3p

TGTAGTGTTTCCTACTTTATGG

>gac-miR-142-5p

CATAAAGTAGAAAGCACTACT

>gac-miR-143-3p

TGAGATGAAGCACTGTAGCTC

>gac-miR-143-5p

GGTGCAGTGCTGCATCTCTGGTC

>gac-miR-144-3p

CTACAGTATAGATGATGTAC

>gac-miR-144-5p

GGATATCATCTTATACTGTAAGT

>gac-miR-145-3p

GGATTCCTGGAAATACTGTTCT

>gac-miR-145-5p

GTCCAGTTTTCCCAGGAATCCC

>gac-miR-146a-3p

ATCTATGGGCTCAGTTCTTTTG

>gac-miR-146a-5p

TGAGAACTGAATTCCATAGATGG

>gac-miR-147-3p

CTTTGCGTTGACACTCGG

>gac-miR-147-5p

ACAGAATCCTTTCTGCACACA

>gac-miR-148a-3p

TCAGTGCATTACAGAACTTTGT

>gac-miR-148a-5p

AAGTTCTGTGAAACACTCCGACT

>gac-miR-150-3p

CGCTGGACAGGTTTGGGGGGGG

>gac-miR-150-5p

ACTCCCAATCCTTGTACCAGTG

>gac-miR-152a-3p

TCAGTGCATAACAGAACTTTGC

>gac-miR-152a-5p

AAGTTCTGTGATACACTCTGACT

>gac-miR-153a-3p

TTGCATAGTCACAAAAGTGATC

>gac-miR-153a-5p

TCATTTTTGTGATGTTGCAGCT

>gac-miR-153b-3p

TTGCATAGTCACAAAAATGATC

>gac-miR-153b-5p

TCATTCTTGTGGTTTGCAACT

>gac-miR-153c-3p

TTGCATAGTCACAAAAATGAGC

>gac-miR-153c-5p

GTCATTTTTGTGGTTTGCAGCT

>gac-miR-155-5p

TTAATGCTAATCGTGATAGGGGT

>gac-miR-15a-2-3p

CAGGCCATGCTGTGCTGCCACA

>gac-miR-15a-2-5p

TAGCAGCACGGAATGGTTTGT

>gac-miR-15b-1-3p

CAAATCATTTTGTGCTGCCACC

>gac-miR-15b-1-5p

TAGCAGCACATCATGTTTTGC

>gac-miR-15c-3p

TGCGAACCATTATTTGCTGCTT

>gac-miR-15c-5p

TAGCAGCGCATCATGGTTTGA

>gac-miR-16a-3p

CCCAATATTAGCAGTGCTGCTT

>gac-miR-16a-5p

TAGCAGCACGTAAATATTGGC

>gac-miR-16c-3p

TCCAATATTGATCGTGCTGCTG

>gac-miR-16c-5p

TAGCAGCACGTAAATATTGGA

>gac-miR-1788-3p

CAGGCAGCTAAAGCAAGTCT

>gac-miR-1788-5p

GGCTTGTTTTAAGTTGCCTGCG

>gac-miR-17a-1-3p

CTGCAGTGGAGGCACTTACAGC

>gac-miR-17a-2-3p

CTGCAGTGAAGGCACTTTCAGC

>gac-miR-17a-5p

CAAAGTGCTTACAGTGCAGGTAG

>gac-miR-181a-1-3p

ACCATCGACCGTTGATTGTACC

>gac-miR-181a-2-3p

ACCATCGACCGTTGACTGTACC

>gac-miR-181a-3-3p

ACCATCGACCGTTGACTGTGCC

>gac-miR-181a-5-3p

ACCACCGAGTGCTGAGTGTACC

>gac-miR-181a-5p

AACATTCAACGCTGTCGGTGAG

>gac-miR-181b-1-5p

AACATTCATTGCTGTCGCTGGGT

>gac-miR-181b-2-3p

CTCACTGATCAATGAATGCAGA

>gac-miR-181b-2/3-5p

AACATTCATTGCTGTCGGTGGGT

>gac-miR-181b-3-3p

CTCACTGAACGATGAATGCAA

>gac-miR-181c-5p

CACATTCATTGCTGTCGGTGGGT

>gac-miR-182a-3p

TGGTTCTAGACTTGCCAACT

>gac-miR-182a-5p

TTTGGCAATGGTAGAACTCACA

>gac-miR-182b-5p

TTTGGCAATGGTAGAACTCACT

>gac-miR-183-5p

TATGGCACTGGTAGAATTCACT

>gac-miR-183a-3p

TGAATTACCATAGGGCCATAA

>gac-miR-184-3p

TGGACGGAGAACTGATAAGG

>gac-miR-184-5p

CCTTATCACTTTTCCAGCCCAGC

>gac-miR-18a-3p

ACTGCCCTAAGTGCTCCTTCT

>gac-miR-18a/b-5p

TAAGGTGCATCTAGTGCAGA

>gac-miR-18b-3p

ACTGCCCTAAGTGCCCCTTCT

>gac-miR-18c-5p

TAAGGTGCATCTAGTGTAGTCA

>gac-miR-190a-3p

ACTATATATCAAACATATTCCT

>gac-miR-190a-5p

TGATATGTTTGATATATTAGG

>gac-miR-190b-5p

TGATATGTTTGATATTCGGTTG

>gac-miR-191-3p

GCTGGTTACGGGGTCCGTTTCT

>gac-miR-191-5p

TAACGGAACCCATAATGCAGCT

>gac-miR-192-3p

CCTGTCAGTTCTCTAGGCCACT

>gac-miR-192-5p

ATGACCTATTAATTGACAGCC

>gac-miR-193a-2-3p

AACTGGCCTACAAAGTCC

>gac-miR-193a-2-5p

TGGGTCTTTGCGGGCAAGGTGA

>gac-miR-194a-3p

CCAGTGGAGGTGCTGTTACCTG

>gac-miR-194a-5p

TGTAACAGCAACTCCATGTGGA

>gac-miR-194b-5p

TGTAACAGCATCTCCATATGGA

>gac-miR-196a-2-3p

CTGCAACACGAAACTGTCTTA

>gac-miR-196a-5p

TAGGTAGTTTCATGTTGTTGGG

>gac-miR-196d-3p

CCACAACACGAAACTGCCTTGA

>gac-miR-196d-5p

TAGGTAGTCTCATGTTGTTGGGC

>gac-miR-199-3p

ACAGTAGTCTGCACATTGGTTA

>gac-miR-199-5p

CCCAGTGTTCAGACTACCTGTTC

>gac-miR-19a-1-3p

TGTGCAAATCTATGCAAAACT

>gac-miR-19a-1-5p

GCTAGTTTTGCATAGTTGCACT

>gac-miR-19a-2-3p

TGTGCAAATCTATGCAAAGCT

>gac-miR-19a-2-5p

AGTTTTGCATAGTTGCGCTCC

>gac-miR-19b-1-5p

AGTTTTGCTGGTTTGCATCCAGC

>gac-miR-19b-2-5p

AGTTTTGCTGGTTTGCTTTC

>gac-miR-19b-3p

TGTGCAAATCCATGCAAAACT

>gac-miR-19c-3p

TGTGCAAATCCATGCAAAGCTCT

>gac-miR-19c-5p

AGTTTTGTTGGTTTGCTTTCAGC

>gac-miR-200a-3p

TAACACTGTCTGGTAACGATGT

>gac-miR-200a-5p

CATCTTACCCGACAGTGCTGGA

>gac-miR-200b-3p

TAATACTGCCTGGTAATGATGA

>gac-miR-200b-5p

CATCTTACGAGGCAGCATTGGA

>gac-miR-202-3p

AGAGGTATAGGGCATGGGAAA

>gac-miR-202-5p

TTCCTATGCATATACCTTTTTC

>gac-miR-204-1-3p

GGCTGGGAAGGCAAAGGGACGC

>gac-miR-204-1/2b-5p

TTCCCTTTGTCATCCTATGCCT

>gac-miR-204-2b-3p

GCAGGGACAGCAAAGGGATGC

>gac-miR-205-1-5p

TCCTTCATTCCACCGGAGTCTG

>gac-miR-205-1a-3p

GATTTCAGTGGTGTGAAGTGTA

>gac-miR-205-1b-3p

GATTTCAGTGGTTTGAAGAGTA

>gac-miR-206-1-3p

TGGAATGTAAGGAAGTGTGTGG

>gac-miR-20a-1-3p

ACTGCAATATAAGCACTTGAA

>gac-miR-20a-2-3p

ACTGCAGTGTGAGCACTTGAAA

>gac-miR-20a-5p

TAAAGTGCTTATAGTGCAGGTAG

>gac-miR-20b-5p

CAAAGTGCTCACAGTGCAGGTAG

>gac-miR-21-5p

TAGCTTATCAGACTGGTGTTGGC

>gac-miR-210-3p

CTGTGCGTGTGACAGCGGCT

>gac-miR-210-5p

AGCCACTGACTAACGCACATTG

>gac-miR-212-3p

TAACAGTCTACAGTCATGGCT

>gac-miR-212a-5p

ACCTTGGCTCTAGACTGCTTACT

>gac-miR-212b-5p

ACCTTGGCTTTAGACTGCTTACT

>gac-miR-214a-3p

TACAGCAGGCACAGACAGGC

>gac-miR-214a-5p

TGCCTATCTACACTTGCTGTGC

>gac-miR-216a-1-3p

CACAATGGCCTCTGGGATTATG

>gac-miR-216a-1-5p

AAATCTCAGCTGGCAACTGTGA

>gac-miR-216a-2-5p

TAATCTCAGCTGGCAACTGTGA

>gac-miR-216b-3p

ACAATGACCTGGAGAGATTCT

>gac-miR-216b-5p

TAATCTCTGCAGGCAACTGTGA

>gac-miR-217a-3p

CAACAGTACCTGATGCATTGCC

>gac-miR-217a-5p

TACTGCATCAGGAACTGATTGGC

>gac-miR-217b-5p

TACTGCATCAGGAACTGAATGG

>gac-miR-2184-3p

GCACATGAGCTTTTACGGTTAA

>gac-miR-2184-5p

AACAGTAAGAGTTTATGTGC

>gac-miR-2187-3p

TTTACAGGCTATGCTAATCT

>gac-miR-2187a-5p

TTAATTAGTATAGCCTGTTTT

>gac-miR-2188-3p

GCTGTGTGAGGTCAGACCTATC

>gac-miR-2188-5p

AAGGTCCAACCTCACATGTCC

>gac-miR-218a-2-3p

AACATGGTTCTGTCAAGCACC

>gac-miR-218a-2-5p

TTGTGCTTGATCTAACCATGTGT

>gac-miR-218b-5p

TTGTGCTTGATCTAACCATGC

>gac-miR-219-1-3p

AGAATTGTGTATGGACATCTGT

>gac-miR-219-2-3p

AGAATTGTGCCTGGACATCTGT

>gac-miR-219-3-3p

GGAGTTGTGGATGGACATCACGC

>gac-miR-219-4-3p

GGAGTTGTGGATGGACATCATGC

>gac-miR-219-5p

TGATTGTCCAAACGCAATTCT

>gac-miR-21a-3p

CGACAACAGTCTGTAGGCTGT

>gac-miR-221-3p

AGCTACATTGTCTGCTGGGTTT

>gac-miR-221a-5p

ACCTGGCATACAATGTAGATTT

>gac-miR-221b-5p

ACCTGGCATTCAGTGTAGGATTCTGT

>gac-miR-222-3p

AGCTACATCTGGCTACTGGGTCT

>gac-miR-222a-5p

TGCTCAGTAGGCAGTGTAGATCC

>gac-miR-222c-5p

TGCTCAGTAGTCAGTGTAGATC

>gac-miR-223-3p

TGTCAGTTTGTCAAATACCCC

>gac-miR-223-5p

TGTATTTGACAAGCTGAGTTGG

>gac-miR-22a-3p

AAGCTGCCAGCTGAAGAACT

>gac-miR-22a-5p

AGTTCTTCACTGGCAAGCTTT

>gac-miR-22b-3p

AAGCTGCCAGTTGAAGAGCTGT

>gac-miR-22b-5p

CGTTCTTCACTGGCTAGCTTT

>gac-miR-23a-1-5p

GGGTTCCTGGCACCGTGATTT

>gac-miR-23a-2-5p

GGAATTCCTGGCAGAGTGATTT

>gac-miR-23a-3-5p

AGGATTCCTGGCAGAGTGATTT

>gac-miR-23a-3p

ATCACATTGCCAGGGATTTCC

>gac-miR-23b-2-3p

ATCACATTGCCAGGGATTACC

>gac-miR-23b-2-5p

GGGTTCCTGGCGTGCTGATTT

>gac-miR-24-2-5p

TGCCTGCTGTGCTGATAATC

>gac-miR-24-3-5p

TGCCTACTGAGCTGATAATC

>gac-miR-24-3p

TGGCTCAGTTCAGCAGGAAC

>gac-miR-24-4a-5p

TGCCTACTGAACTGGTATCAGT

>gac-miR-24-4b-5p

TGCCTACTGAACTGGATTCAGT

>gac-miR-24-6-5p

TGCCTACTGAGCTGATAACAGT

>gac-miR-26-5p

TTCAAGTAATCCAGGATAGG

>gac-miR-26a-1a-3p

CCTATTTGGGATGACTTGGTTC

>gac-miR-26a-1b-3p

CCTATTCCGGATGACTTGGTTC

>gac-miR-26a-2-3p

CCTACTCGTGATTACTTGCACT

>gac-miR-26a-3-3p

TGGCCTGTGCTTGATTACTTGC

>gac-miR-26b-3p

CCTATTCTTGATTACTTGTTTC

>gac-miR-27a-3p

TTCACAGTGGCTAAGTTCCGC

>gac-miR-27a-5p

AGGACTTAGCTCGCTCCGTGA

>gac-miR-27b-3p

TTCACAGTGGCTAAGTTCTGC

>gac-miR-27b-5p

AGAGCTTAGCTGATTGGTGAAC

>gac-miR-27c-1-3p

TTCACAGTGGTTAAGTTCTGC

>gac-miR-27c-1-5p

AGGACTTAACCCACATGTGAGCA

>gac-miR-27e-1-5p

AGAGCTTAGCTAATTGGTGAGC

>gac-miR-27e-3p

TTCACAGTGGCTAAGTTCAGT

>gac-miR-29a-1-5p

ACCGATTTCTTCTGGTGCATAGA

>gac-miR-29a-3a-5p

ACCGATTACTTTTGGTGTTCAGA

>gac-miR-29a-3b-5p

ACTGGTTTCAGATGGTGTCTTAGA

>gac-miR-29a-3p

TAGCACCATTTGAAATCGG

>gac-miR-29b-1a-5p

CTGGTTTCAGATGGTGGCTTAGA

>gac-miR-29b-1a/2a-3p

TAGCACCATTTGAAATCAGT

>gac-miR-29b-1b-3p

TAGCACCATTCGAAATCGGTT

>gac-miR-29b-1b-5p

ACCGCTTTCGTGTGGTGTTCAGA

>gac-miR-29b-2a-5p

CTGGTTTCATATGGTGGTTTAGA

>gac-miR-29b-2b-3p

CTAGCACCATATGAAATCAGTG

>gac-miR-29b-2b-5p

CTGATTTCATTGGGTGAGGTAGA

>gac-miR-301a-3p

CAGTGCAATAGTATTGTCAAAGC

>gac-miR-301a-5p

GCTCTGACTTCATTGCACTACT

>gac-miR-301b-5p

GCTTTGACGATGTTGCACTACT

>gac-miR-301b/c-3p

CAGTGCAATAGTATTGTCATAGC

>gac-miR-301c-5p

CTTTGACAATGTTGCACTACT

>gac-miR-30a-3p

CTTTCAGTCCGATGTTTGCTGC

>gac-miR-30a-5p

TGTAAACATCCCCGACTGGAA

>gac-miR-30bb-3p

CTGAGAGAAGGTTGTTTACTTG

>gac-miR-30bb-5p

TGTAAACATCCTACACTCAGCT

>gac-miR-30c-1-3p

CGGGAGTGGGAATGTTTATACT

>gac-miR-30c-1-5p

TGTAAACATCCTACACTCTCGG

>gac-miR-30c-2-3p

CTGGGAGAAGGGTGTTTACTCT

>gac-miR-30c-2-5p

TGTAAACATCCTACACTCTCAGC

>gac-miR-30e-3p

CTTTCAGTCGGATGTTTGCAGC

>gac-miR-30e-5p

TGTAAACATCCTTGACTGGAAGCT

>gac-miR-3120-3p

TGCACAGCAAGTGTAGATAGGC

>gac-miR-3120-5p

CTGTCTGTGCCTGCTGTACAGG

>gac-miR-31a-5p

AGGCAAGATGTTGGCATAGCTG

>gac-miR-33-5p

GTGCATTGTAGTTGCATTGC

>gac-miR-338-1a-5p

AACAATATCCTGGTGCTGCCTGAGT

>gac-miR-338-1b-5p

AACAACATCCTGGTGCTGCCTGAGT

>gac-miR-338-3p

TCCAGCATCAGTGATTTTGTT

>gac-miR-33a-3p

CAATGTGTCTGCAGTGCAGTA

>gac-miR-33ba-3p

CAATGCACCTGCAGTGCAAC

>gac-miR-33bb-3p

CAATGGATCTCCTCTGCAACA

>gac-miR-34a-3p

AATCAGCAAGTATACTGCCGCA

>gac-miR-34a-5p

TGGCAGTGTCTTAGCTGGTTGT

>gac-miR-3618-5p

TGATTTCCAATAATTGAGACAGT

>gac-miR-365-2b-3p

TAATGCCCCTAAAAATCCTTAT

>gac-miR-365-2b-5p

AGGGACTTTTAGGGGCAGCTGTG

>gac-miR-375a-3p

TTTGTTCGTTCGGCTCGCGTTA

>gac-miR-375a-5p

ACGTTGAGCCACACGTACAATAC

>gac-miR-425-3p

AACCGGGAACTTCGTGTCAGC

>gac-miR-425-5p

AATGACACGTTTTCTCCCGGATT

>gac-miR-429a-3p

TAATACTGTCTGGTAATGCCG

>gac-miR-449-5p

AGGCAGTGTCTTGTTAGCTGG

>gac-miR-451-5p

AAACCGTTACCATTACTGAGT

>gac-miR-454b-3p

TAGTGCAATATTGCTTATAGGGT

>gac-miR-455-3p

TGCAGTCCATGGGCATATACAC

>gac-miR-455-5p

TATGTGCCCTTGGACTACATCG

>gac-miR-456-3p

CAGGCTGGTTAGATGGTTGTC

>gac-miR-458-3p

ATAGCTCTTTAAATGGTACTGC

>gac-miR-458-5p

AGCGCCATTTTCAGAGCTATA

>gac-miR-459-5p

TCAGTAACAAGGATTCATCCT

>gac-miR-460-3p

CACAGCGCATACAATGTGGA

>gac-miR-460-5p

CCTGCATTGTACACACTGTGC

>gac-miR-489-3p

GTGACATCATATGTACGGCTGC

>gac-miR-489-5p

TGGTCGTATGTATGACGTCATT

>gac-miR-499a-3p

AACATCACTCTAAGTCTGTGCT

>gac-miR-499a-5p

TTAAGACTTGCAGTGATGTTT

>gac-miR-551a-3p

GCGACCCATCCTTAGTTTCTGA

>gac-miR-551a-5p

GAAACTAAGCGTGGGTGTGGCCT

>gac-miR-7-5p

TGGAAGACTAGTGATTTTGTTG

>gac-miR-7132a-3p

TGGGGAGCTTAGAACAAGTGT

>gac-miR-7132a-5p

GACTTGGTCAAAGCTCCTCAGT

>gac-miR-7132b-3p

TGAGGCGTTTGGAACAAGTTC

>gac-miR-7132b-5p

GACTTGGTCAAAGCTCCTCAGC

>gac-miR-7133-3p

TAGTTTGATACACAGCACAAT

>gac-miR-7133-5p

GATGTTGAGTGTCAAACTGTAT

>gac-miR-7147-3p

TGGTTGACCAGTGTTGTGCCTC

>gac-miR-7147-5p

TGTACCATGCTGGTAGCCAGT

>gac-miR-722-3p

TTTTGCAGAAACGTTTCAGATT

>gac-miR-722a-5p

TGAAACGTTTTCGCCAAAAATG

>gac-miR-722b-5p

TTGAAACGTTTTAGCCAAAAA

>gac-miR-723-3p

AGACATCAGATAAATCTGT

>gac-miR-723b-5p

GGCAGTTTTGATGATGTTACTT

>gac-miR-724-3p

CAGCCACACCTTCCTTTTAAGA

>gac-miR-724-5p

TTAAAGGGAATTTGCGACTGTT

>gac-miR-725-3p

TTCAGTCATTGTTTCTGGTCGT

>gac-miR-725-5p

TGCTGGAAACCTTGCCTGTGAAT

>gac-miR-726-5p

GGAATTCCGCTAGTTCTGAACTAT

>gac-miR-727b-3p

GTTGAGGCGAGTTGAAGACT

>gac-miR-727b-5p

TCAGTCTTCAATTCCTCCCAGC

>gac-miR-728-3p

ATACTAAGTACACTACGTTTA

>gac-miR-728a-5p

AATGTAGTAGACTTAAAGTAT

>gac-miR-728b-5p

AAATGTAGTAGACTATAAGTAT

>gac-miR-730-3p

CACAGCGCCTGCAATGTGGA

>gac-miR-730-5p

TCCTCATTGTGCATGCTGTGTG

>gac-miR-733-1-5p

GCATCGGTTTAGCTCAGTGGTTACTTCC

>gac-miR-733-2-5p

ATCTTTCTCTGACGGGCT

>gac-miR-733-3-5p

CAGTGGTTACTTCTGGT

>gac-miR-733-3p

CGACCACGGGCGCTGTCCG

>gac-miR-734-3p

TAAATGCTGCAGAATTGTGCTC

>gac-miR-734-5p

TGAACTATTCTGCAACATTTGT

>gac-miR-736-1-3p

GTAAGACGAACAAAAAGTTTGT

>gac-miR-736-1-5p

CAGCTTTTTGTTTGTGTTATGT

>gac-miR-737-5p

GTTTTTTTAGGTTTTGATTTT

>gac-miR-7552a-5p

TTACAATTAAAGGATATTTCTC

>gac-miR-7a-1/3-3p

CAACAAGTCCCAGTCTACCTC

>gac-miR-7a-4-3p

CAACAAGTCATTGTCTCCCGCG

>gac-miR-7bb-3p

CAACAAATCACAGTCTGCCA

>gac-miR-8159-5p

TCAGTAACTGGAATCTGTCCCT

>gac-miR-8160a-5p

AGAATAATGCCAGCAGTCGGTC

>gac-miR-8160b-3p

CCAGCGCTGGTGTTATTGAGT

>gac-miR-8160b-5p

AGAATAATGCCAGCAGTCGGCC

>gac-miR-9-1/3/5/6-3p

ATAAAGCTAGATAACCGAAAGT

>gac-miR-9-5p

TCTTTGGTTATCTAGCTGTAT

>gac-miR-9-7-3p

TAAAGCTAGAGAACCGAAAGT

>gac-miR-92a-1-5p

AGGTGGGGATTAGTAGCAATGCT

>gac-miR-92a-2-5p

AGGTTGGGAGAGGTAGCAATGCT

>gac-miR-92a-3p

TATTGCACTTGTCCCGGCCTGT

>gac-miR-96-5p

TTTGGCACTAGCACATTTTTGC

>gac-miR-96a-3p

CAATTATGTGTAGTGCCAATAT

>gac-miR-99a-3p

CAAGCTCGCCTCTGTGGGTCT

>gac-miR-99a-5p

AACCCGTAGATCCGATCTTGT

>gac-miR-ENSGACG00000022167-3p

GCTTCAAACCTGTAGCTGCT

>gac-miR-ENSGACG00000022167-5p

GCCCGGATGAGCCTCGGTGGT

>gac-miR-Rastorguev366-3p

CTAACGTTAGTGTCGCTGGAC

>gac-miR-Rastorguev366-5p

CCCGGCGAAGTAACGTTAGCT

>gac-miR-Rastorguev443-3p

TGTGTCCCAATTTCAGACCGG

>gac-miR-Rastorguev443-5p

GGTCAAAGGTTTGGACACACG

>gac-miR-Rastorguev458-3p

CTGAGGTACCAGAACGCCGGCCC

>gac-miR-Rastorguev458-5p

CGCTGCGTTCGGTGCCTCGTCC

Supplementary File 7: miRNA isomiR diversity in stickleback and zebrafish.


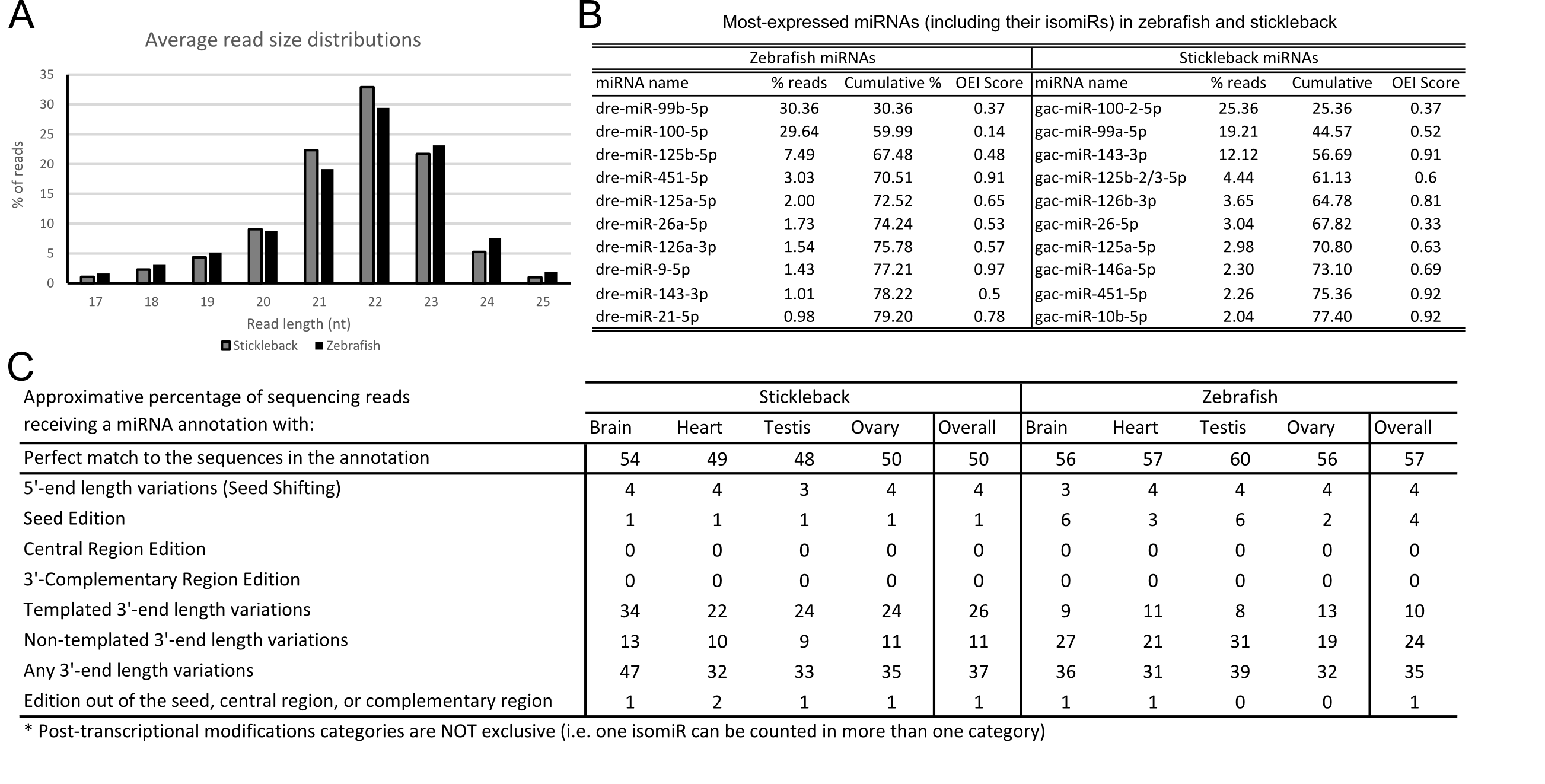


Supplementary Table 1: small RNA library sequencing statistics.

Supplementary Table 2: PCR primers used for the study of miR2188-5p.
